# Temperature‐Induced Morphological Transitions for On‐Demand Detachment in Worm‐Based Polymer Hydrogels

**DOI:** 10.1002/anie.202519031

**Published:** 2025-11-17

**Authors:** Kaixiang Yang, Julia Y. Rho, Laihui Xiao, Megan R. Elliott, Calum T. J. Ferguson, Yezi You, Rachel K. O'Reilly

**Affiliations:** ^1^ School of Chemistry University of Birmingham Edgbaston Birmingham B15 2TT UK; ^2^ CAS Key Laboratory of Soft Matter Chemistry Chinese Academy of Science Department of Polymer Science and Engineering University of Science and Technology of China Hefei Anhui 230026 China

**Keywords:** Morphology transition, On‐demand detachment, PISA, RAFT polymerisation, Temperature responsive adhesive

## Abstract

Temperature‐responsive materials are vital for applications relating to on‐demand detachable adhesives, as they enable rapid, damage‐free detachment. However, low‐temperature responsive systems often lack pronounced phase change, which can lead to undesirable mechanical interlocking with complicated substrate interfaces. Polymerisation‐induced self‐assembly (PISA) has been effectively employed to fabricate worm‐like hydrogels exhibiting temperature‐induced phase transitions; however, its application in on‐demand detachment remains underexplored. Building on this, we leverage precise morphological tuning across a broad temperature range (1 to 70 °C) to modulate the hydrogel phase. Our hydrogel exhibits a reversible transition from a robust adhesive state at room temperature to a non‐adhesive liquid phase upon both cooling and heating. This approach overcomes the limitations associated with low‐ and high‐temperature responsive detachment on complex surfaces, thereby broadening its utility.

## Graphical abstract

We developed a temperature‐responsive adhesive hydrogel via polymerisation‐induced self‐assembly (PISA), where adhesion strength is modulated by temperature‐driven morphological transitions resulting core block hydrophilicity change. This system enables detachment at both high and low temperatures—particularly on complex surfaces—while enhancing the hydrogel's reusability and recyclability.

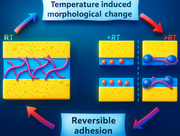

Adhesives play a critical role in both biological and industrial applications,^[^ ^1^, ^2^, ^3^, ^4^ ^]^ especially in advanced systems such as sensors,^[^ ^5^, ^6^ ^]^ wound patches^[^ ^7^, ^8^, ^9^ ^]^ and biomedical devices that require on‐demand detachable adhesion.^[^ ^10^ ^]^ Their performance is governed by two primary factors: the interfacial adhesion between the adhesive and the substrate and the internal cohesion of the adhesive materials.^[^ ^11^ ^]^ While altering the interfacial adhesion typically involves complex and expensive surface modifications, tuning the internal cohesion offers a more practical and versatile alternative. By incorporating functional groups or additives that respond reversibly to external stimuli—such as light,^[^ ^12^ ^]^ pH^[^ ^13^ ^]^ or temperature^[^ ^14^ ^]^—the adhesive's internal cohesion can be dynamically adjusted through phase transitions, intermolecular interactions and polymer chain entanglement.^[^ ^15^ ^]^ This strategy not only enables the efficient switching of adhesion but also broadens and enhances performance in diverse environments.

Of all the stimuli‐responsive adhesives, high‐temperature responsive detachable materials are widely utilised due to their rapid, cost‐effective and energy‐efficient response.^[^ ^16^ ^]^ However, elevated temperature can lead to thermal degradation of sensitive substrates, including skin, food packaging, or battery components. Low‐temperature detachable adhesives offer a gentler alternative. Here, cooling increases intermolecular interactions or induces crystallisation, thereby restricting the mobility of polymer chains and enhancing internal interactions. This increases cohesion and renders the adhesive more rigid, thus reducing interfacial adhesion strength.^[^ ^17^, ^18^, ^19^ ^]^ For example, Lu and coworkers demonstrated that at body temperature (37 °C), entangled gelatin methacryloyl chains have strong adhesion due to exposed reactive groups. Upon cooling, intermolecular hydrogen bonds (H‐bonds) form within the material, reducing surface interactions.^[^ ^17^ ^]^ However, mechanical interlocking of the polymer adhesive with complex surfaces (e.g., sponges) can inhibit the detachment at low temperature. Moreover, this technique requires complex and costly surface modifications to create the ideal surfaces.^[^ ^20^ ^]^

To date, homogeneous adhesives have rarely demonstrated dual‐temperature detachment capability—a feature that would greatly enhance its utility, practicality and commercial potential. Recent advances in polymerisation‐induced self‐assembly (PISA) have identified polymeric nanostructures as promising gelators due to their functional and responsive architectures.^[^ ^21^, ^22^, ^23^, ^24^, ^25^, ^26^ ^]^ Hydrogels prepared via PISA have demonstrated thermally reversible phase changes; these temperature‐responsive diblock polymers are capable of forming morphologies including spheres, worms, lamellas, or vesicles that can thermally transition to different states, as characterised using techniques including small‐angle X‐ray scattering (SAXS).^[^ ^27^, ^28^, ^29^, ^30^, ^31^, ^32^, ^33^ ^]^ This enables facile temperature‐controlled gelation and sterilisation. Worm‐based hydrogels can be produced to exhibit advantageous temperature‐dependent phase change behaviour.^[^ ^34^ ^]^ However, due to their low modulus and cohesion, they are currently unsuitable for use in adhesive materials.

Here, we present a one‐step synthesis of temperature‐responsive on‐demand detachable worm‐based hydrogel formed using PISA. In this system, hydroxyl (─OH) groups in the corona block secure interfacial adhesion, while the worm chains enhance internal cohesion through entanglement and hydrogen bonding, resulting in a strong modulus. Both of the above interactions enable tunable adhesion over a broad temperature range, achieving on‐demand detachment through morphology‐induced phase transitions upon both heating and cooling. This not only combines the advantages of both high and low temperature responsive detachment for adhesive materials, but also expands the application potential of PISA‐based materials. These synergistic properties render the material a great candidate for uses in food storage monitoring, robotic grasp‐and‐release mechanisms, surgical wound healing and environmental temperature sensing.^[^ ^19^, ^35^, ^36^, ^37^ ^]^

The target polymers were synthesised using RAFT‐PISA. First, the hydrophilic macromolecular chain transfer agent (macro‐CTA) PHEAm_49_ was synthesised by using 2‐(butylthiocarbonothioylthio) propanoic acid (PABTC) as a chain transfer agent and *N*‐hydroxyethyl acrylamide (HEAm) as a monomer (Table S1, Figures S1,2). To access higher‐order morphologies, the hydrophilic HEAm was incorporated into the hydrophobic diacetone acrylamide (DAAm) block, similar to the previously reported mechanism.^[^ ^38^ ^]^ Then, the macro‐CTA is chain extended to yield PHEAm_49_‐*b*‐(PHEAm_36_‐*co*‐PDAAm_115_), which was carried out at high solids content (42.8 wt%, Figure 1a). During polymerisation, the polymers self‐assemble into spheres and then assemble into worm chains as the degree of polymerisation (DP) increases. Due to the entanglements and H‐bonds of the worm chains, they function as physical crosslinkers, forming a free‐standing worm‐based hydrogel network (Figure S3). High conversion was achieved and was confirmed through ^1^H NMR spectroscopy (conversions > 99%, Figure S4). SEC (Size exclusion chromatography) analysis was then carried out and indicated control of molecular weight for this diblock copolymer, with a relatively narrow molecular weight distribution (*M*_w_/*M*_n_ = 1.12, Table S2). Polymers with different lengths and weight ratios were prepared to determine the effect of varying the DPs and solid content on cohesion and adhesion, as shown in Figures S5–S11 and Table S2.

**Figure 1:**
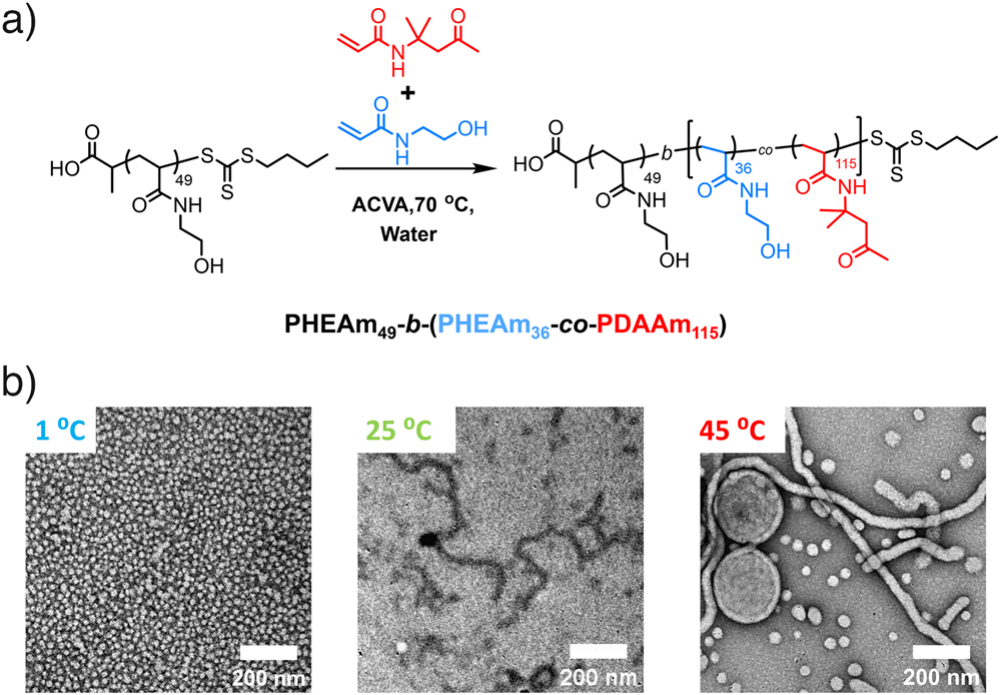
a) PISA by using macro‐CTA (PHEAm_49_), DAAm and HEAm to yield the amphiphilic block copolymer PHEAm_49_‐*b*‐(PHEAm_36_‐*co*‐PDAAm_115_). b) TEM images illustrating the self‐assembled morphologies of PHEAm_49_‐*b*‐(PHEAm_36_‐*co*‐PDAAm_115_) at 1, 25 and 45 °C, with a weight ratio of 42.8 wt.%.

The system exhibits reversible morphological transitions among three distinct states: spheres at 1 °C, worms at room temperature (RT, 25 °C), and vesicles at 45 °C, corresponding to transitions from a liquid phase to a solid hydrogel and finally to a liquid phase again. As the internal cohesion of the hydrogel changes in response to these phase transitions, its adhesion strength is also modulated. Strong adhesion is maintained at RT, while both heating and cooling lead to weak adhesion and trigger detachment (Figure S12). Transmission electron microscopy (TEM, sample prepared with [O, O´‐1,3‐propanediylbishydroxylamine dihydrochloride (O‐alkyl hydroxylamine) as crosslinker), confirmed that at RT, the hydrogel displays a worm morphology that underpins its hydrogel network (Figure 1b). Storage at 45 °C produces a mixed phase of vesicles and worms, whereas storage at 1 °C results in spheres.^[^ ^39^ ^]^ Variable temperature ^1^H NMR spectroscopy was employed to investigate the temperature‐dependent changes in the polymer's solvation. A 10 w/w% aqueous hydrogel dispersion was heated from 10 to 70 °C, with spectra recorded at 10 °C intervals and normalised relative to the external standard pyridine. The ^1^H NMR signal corresponding to the core‐forming P(HEAm_36_‐*co*‐DAAM_115_) blocks became more prominent at higher temperatures, indicating increased hydration of this hydrophobic structure‐directing block (Figure S13). This finding aligns with the observation that the core block becomes more hydrophobic as the temperature decreases, leading to a transition from a worm‐like structure to a spherical morphology. Conversely, increasing the temperature promotes the transition from worms to vesicles, attributed to the enhanced hydration of the core block, facilitating the transition to a higher structural morphology.^[^ ^27^ ^]^

Rheological studies further elucidated these morphological transitions and gelation behaviours. At 10 °C, the system behaves as a viscous liquid dominated by free‐flowing spheres, with the loss modulus (G″) exceeding the storage modulus (G″), indicating a gel–sol transition (Figure 2a). At RT, worm chain entanglements form a robust, free‐standing hydrogel (G’ > G″), whereas further heating results in a marked decrease in modulus and increased turbidity—consistent with a second gel–sol transition—yet G’ remains higher than G″ from 25 to 70 °C, reflecting partial‐retention of the hydrogel state. TEM imaging confirms a mixed phase of worms and vesicles, implying that some worms remain entangled while others transition into vesicles, a finding corroborated by viscosity trends.

**Figure 2:**
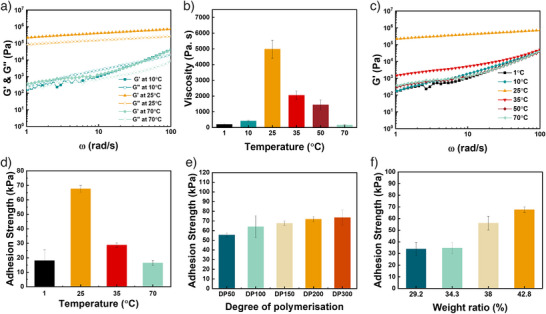
a) The storage modulus and loss modulus of PHEAm_49_‐*b*‐(PHEAm_36_‐*co*‐PDAAm_115_) at different temperatures (10, 25 and 70 °C) and a set weight ratio of 42.8 wt.%. b) The storage modulus of PHEAm_49_‐*b*‐(PHEAm_36_‐*co*‐PDAAm_115_) from 1 to 70 °C, at 42.8 wt.%. c) The viscosity of PHEAm_49_‐*b*‐(PHEAm_36_‐*co*‐PDAAm_115_) from 1 to 70 ⁰C and a set weight ratio of 42.8 wt.%. d) Adhesion strength of PHEAm_49_‐*b*‐(PHEAm_36_‐*co*‐PDAAm_115_) from 1 to 70 °C and 42.8 wt. %. e) Adhesion strength of polymers with different core block DPs (50, 100, 150, 200, 300) at 25 °C and 42.8 wt.%. f) Adhesion strength of hydrogel of PHEAm_49_‐*b*‐(PHEAm_36_‐*co*‐PDAAm_115_) at different weight ratios (29.2%, 34.3%, 38%, 42.8% at 25 ⁰C) under 25 °C. All the samples are tested for at least four replicates.

The viscosity and maximum modulus were observed at 25 °C (Figures 2b,c, S14), indicating that the cohesion strength is strongest at RT, which can result in the highest adhesion strength. The adhesion strength of the hydrogel from 1 to 70 °C was then examined (Figure 2d). As the temperature varied, the adhesion strength at low temperatures (1 °C) and high temperatures (70 °C) was 18 and 16 kPa, respectively. At 25 °C, the adhesion strength was significantly higher, up to 70 kPa. Therefore, a dramatic change in adhesion strength of 73% and 76% was observed in cooling and heating, respectively, similar to the state‐of‐the‐art solely high‐temperature responsive adhesive hydrogels.^[^ ^18^, ^40^ ^]^ We further employed atomic force microscopy (AFM) to investigate the nanoscale interfacial adhesion at different temperatures. The highest nanoscale adhesion at room temperature (∼6 nN), decreasing to ∼3 nN at 1 and 45 °C (Figure S15). Supporting the proposed temperature‐dependent detachment mechanism and confirming that the adhesion transition is intrinsic and occurs consistently across both bulk and nanoscale interfaces.

The effect of core block length on adhesion strength was investigated by creating worm‐based hydrogels containing polymers with varying core block DP. All samples were maintained at the same weight ratio (42.8 wt.%), and the tensile adhesion strength was tested as shown in Figure S16. Here, the adhesion strength increased with increasing DP of the core block from 50 to 300 (Figure 2e), from 55 to 73 kPa, with a change of about 18 kPa. However, when the DP was greater than 150, no heating or cooling effect was observed, suggesting the morphological transitions can no longer be achieved. TEM images further show that the worm width at DP 300 was significantly higher than that at DP 150, with average widths of 75 and 24 nm, respectively (Figure S17). This agrees with previous reports that higher core block DP yields longer, thicker worms with greater inter‐worm entanglement on the colloidal scale,^[^ ^41^, ^42^ ^]^ enhancing cohesion and thus adhesion strength. However, excessive DP increases hydrophobicity and reduces water plasticisation, lowering chain mobility and thereby inhibiting phase transitions. Rheological measurements further supported these observations, showing that higher DP corresponds to increased storage modulus (G′) and viscosity, consistent with enhanced worm entanglement (Figures S18a,b).^[^ ^42^ ^]^The weight ratio also influences adhesion strength, as it determines the number of functional groups on the surface of the hydrogel, which in turn affects both the cohesion and adhesion strength. Herein, we investigated the adhesion strength at various weight ratios (29.2, 34.3, 38.0, and 42.8 wt.%) at a fixed core block length (DP = 150). As expected, the adhesion strength increased with the increase in weight ratio (Figure 2f), owing to the greater abundance of polymer chains, which provides a higher concentration of ─OH groups for intra‐network hydrogen bonding as well as enhanced surface interactions with the substrate. Rheological tests further support this trend, showing an enhanced modulus and viscosity with increased weight ratio, which indicates improved cohesion strength (Figures S18c,d). Again, when the weight ratio exceeded 42.8%, the high‐temperature responsive phase transition was not observed due to the increasing entanglements and interaction within the worm chains (Figure S19).

To further investigate the effect of corona H‐bonding interactions and adhesion strength, we varied the functional groups in the hydrophilic corona. We synthesised a PDMA_49_ Marco‐CTA to fabricate a PDMA_49_‐*b*‐(PHEAm_38_‐*co*‐PDAAm_114_) worm‐based hydrogel containing tertiary amine functional groups to compare with the hydroxyl functionality present in PHEAm_49_‐*b*‐(PHEAm_36_‐*co*‐PDAAm_115_) (Figures S20–S22). Rheological and tensile adhesion tests showed increased adhesion strength, modulus, and viscosity for the hydroxyl‐containing HEAm_49_ as corona block (Figure S23), which may be attributed to the successful formation of H‐bonds within the worm chains.^[^ ^43^, ^44^, ^45^ ^]^

In addition to assessing adhesion strength with various DPs and weight ratios, demonstrating the worm‐based hydrogel's versatility and adhesion on different substrates was crucial. Glass substrates were attached to metal, plastic, wood, shell, silica rubber and human skin surfaces (Figure 3a). All the objects maintained firm attachment when the glass plate was kept vertical, indicating the successful formation of strong and versatile adhesions. It is important to note that the adhesive mechanism is mainly due to the formation of H‐bonds from the corona block (PHEAm_49_) to the substrate, such as the hydroxyl groups on glass and tissue or the oxygen on metallic oxide layers. However, these H‐bonds are not the only interaction determining interfacial adhesion. On hydrophobic substrates like plastic or silicone rubber, van der Waals forces and mechanical interlock might become the primary contributors to interfacial adhesion (Figure S24).^[^ ^46^ ^]^

**Figure 3:**
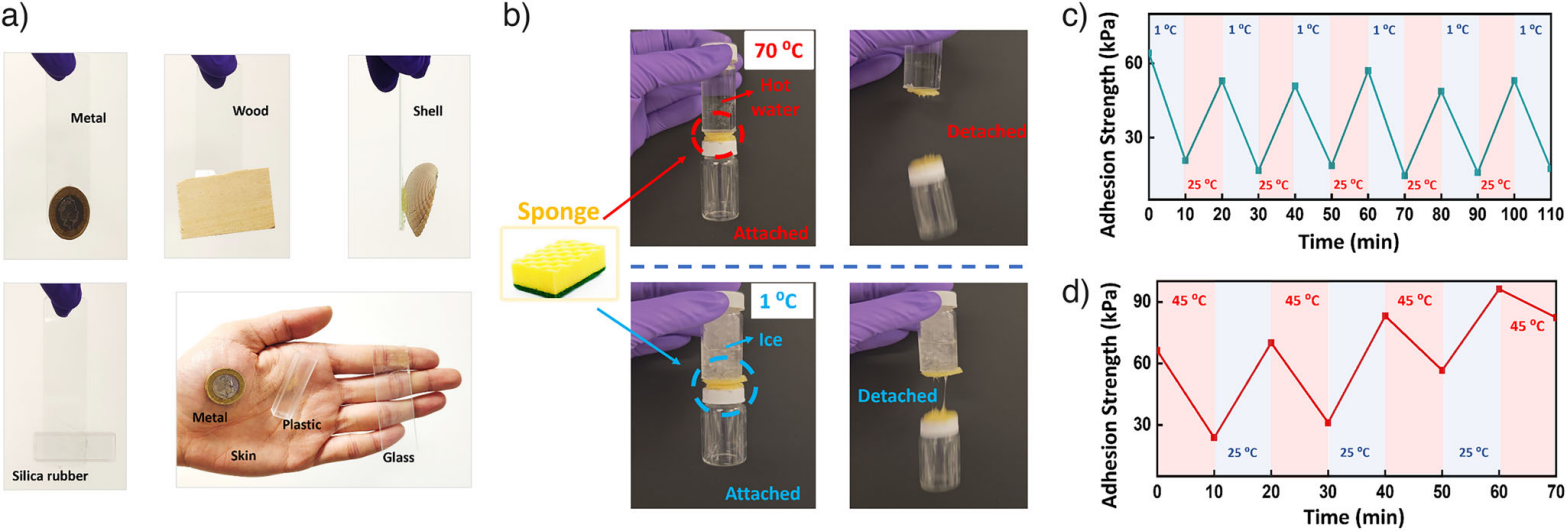
a) The versatile adhesion performance of the hydrogel was shown by attaching it to various substrates (metal, wood, silica rubber, shell, plastic, glass and skin). b) Hydrogel on‐demand detachment from a complicated surface (sponge surface) was triggered by hot water (70 °C) or ice (1 °C) in the upper vial. c) and d) Hydrogel reusability process under temperature modulation, monitoring adhesion strength: (c) repeated attachment–detachment cycles at 1 –25 °C and (d) 25–45 °C for specified durations.

Furthermore, our worm‐based hydrogel exhibits on‐demand detachment through external temperature fluctuations. In one experiment, the hydrogel was used to attach a 104.6 g weight to a glass plate. When subjected to high (70 °C) or low (1 °C) temperatures, the hydrogel transitions to the liquid phase, triggering detachment within 10 s upon heating and approximately 12 s upon cooling (Figure S25). In contrast, a control sample maintained stable adhesion at RT for over 20 min. Notably, similar detachment behaviour was observed on a porous sponge substrate, where mechanical interlocking can cause undesired adhesion (Figure 3b). Specifically, two parts of a commercial sponge were firmly attached to the tops of two vials (21.6 g each) and bonded using 0.2 g of hydrogel across an area of approximately 2 cm^2^. Adding hot water or ice to the upper vial altered the sponge's temperature, inducing a phase change in the hydrogel and resulting in detachment after 33 s (70 °C) and 24 s (1 °C), respectively.

The reversibility of the hydrogel's adhesive properties was evaluated through repeated heating and cooling cycles. Initially, at RT, the hydrogel exhibited an adhesion strength of approximately 62 kPa. However, after cooling for 10 min at 1 °C, the adhesion strength decreased to about 20 kPa, and subsequently returned to ∼60 kPa after 10 min at RT. This reversible switching was maintained over six cycles with no significant loss in performance (Figure 3c). In contrast, at 45 °C, an increase in adhesion strength was observed after each cycle, and the hydrogel could be reused for only three cycles before the adhesion strength ceased to decrease upon heating (Figure 3d). This is likely due to the gradual loss of water upon heating due to evaporation (approximately 5% over 70 min). This decrease in water content increases the solid content of the gel, increasing its adhesive strength but inhibiting the ability to transform between vesicle and worm phases. To further evaluate the durability of the hydrogel, we measured the adhesion strength after 100 attachment–detachment cycles at 1 °C. Here, the performance was maintained with only about a 30% increase in adhesion strength observed, which again we attribute to dehydration of the gel over time (Figure S26). These results indicate that the primary limitation of the system arises from dehydration of the hydrogel matrix rather than chemical degradation of the polymer chains, ultimately leading to the loss of on‐demand detachment and reusability. To mitigate this effect, the hydrogel was dehydrated after extended high‐temperature usage and rehydrated to restore the original weight ratio (42.8 wt.%). The recycled hydrogel demonstrated viscosity, modulus and adhesion strength values similar to those of the freshly prepared gel (Figure S27). TEM images confirmed that the worm‐like nanostructures retained their original morphology and thickness (Figure S28).

We developed an on‐demand detachable hydrogel composed of entangled worm chains. By precisely tuning the DP and mass ratio, we achieved controllable cohesion and reversible temperature‐responsive on‐demand detachment. At RT, the hydrogel exhibits an entangled worm‐like morphology, which transitions to liquid‐like spherical or vesicular structures upon cooling or heating, respectively. These distinct, bidirectional phase transitions enable on‐demand detachment modulated through temperature changes (Figure S29). Comprehensive characterisations—including adhesion tests on various surfaces, thermal cycling and recyclability assessments—demonstrate the material's excellent reversibility and practicality. This work addresses the challenge of low temperature‐responsive detachment on complex surfaces and expands the application potential of PISA‐based smart materials.

## Supporting Information

The authors have provided additional characterisation of the materials synthesised and additional references are cited within the Supporting Information.

## Conflict of Interests

The authors declare no conflict of interest.

## Supporting information



Supporting Information

## Data Availability

The data that support the findings of this study are available from the corresponding author upon reasonable request.
