# Supplementary material for: Temperature‐Induced Morphological Transitions for On‐Demand Detachment in Worm‐Based Polymer Hydrogels
Source: Angew Chem Int Ed Engl. 2025 Nov 17;65(1):e19031. doi: 10.1002/anie.202519031 (PMC12759209; doi:10.1002/anie.202519031)
Supplement: Supplementary file 1 — Supporting Information [file ANIE-65-e19031-s001.docx]

**Temperature-induced morphological transitions for on-demand detachment in worm-based polymer hydrogels**

Kaixiang Yang, ^[a, b]^ Julia Y. Rho,^[a]^ Laihui Xiao,^[a]^ Megan R. Elliott, ^[a]^ Calum T. J. Ferguson,^[a]^ Yezi You,*^[b]^ and Rachel K. O’Reilly*^[a]^

[a] Dr. Kaixiang Yang, Dr. Julia Y. Rho, Laihui Xiao, Dr. Calum T. J. Ferguson, and Prof. Rachel K. O’Reilly
School of Chemistry
University of Birmingham,
Edgbaston, Birmingham, B15 2TT, U.K.

Email: r.oreilly@bham.ac.uk

[b] Dr. Kaixiang Yang, Prof. Yezi You

CAS Key Laboratory of Soft Matter Chemistry, Chinese Academy of Science, Department of Polymer Science and Engineering

University of Science and Technology of China
Hefei, Anhui 230026, China
E-mail: yzyou@ustc.edu.cn

**Supporting Information**

**Materials**

All chemicals were obtained from Sigma-Aldrich, except when specified. The RAFT chain transfer agent (CTA) 2-(butylthiocarbonothioylthio) propanoic acid (PABTC) was synthesized following existing.^[1]^ The thermal initiator used was 4,4′-azobis-4-cyanovaleric acid (ACVA). *N*-Hydroxyethyl acrylamide (HEAm, 97%) was filtered using a basic aluminium oxide (Fisher Scientific) column to remove radical inhibitors. Diacetone acrylamide (DAAm, 99%) was purchased from Alfa Aesar. [O, O´-1,3-propanediylbishydroxylamine dihydrochloride (O-alkyl hydroxylamine crosslinker) was purchased from Sigma Aldrich. Solvents were sourced from different suppliers, including Honeywell, Fisher, and Sigma Aldrich.

**Methods**

**^1^H Nuclear Magnetic Resonance (^1^H NMR) spectroscopy**

NMR spectra were measured using a 400 HD NMR spectrometer, which operated at 400MHz. The residual solvent peaks were used as internal references. ^1^H NMR spectra were recorded in deuterated DMSO using a 400 MHz Bruker Avance III HD 400 spectrometer with 16 scans being averaged per spectrum.

**Variable Temperature ^1^H NMR spectroscopy**

Variable Temperature ^1^H NMR spectra were recorded using a 500 MHz Bruker Avance-500 spectrometer. The freeze-dried PHEAm_49_-*b*-(PHEAm_36_-*co*-PDAAm_115_) diblock copolymer was dispersed in D_2_O at 10% w/w contained with the pyridine standard in. Then allowed the sample to fully dissolve and equilibrate at the desired temperature for 24 hours before recording spectra. Spectra were recorded from 10°C to 70°C at 10°C intervals, with an equilibrium time of 10 minutes at each temperature.

**Size Exclusion Chromatography (SEC)**

We used an Agilent 1260 Infinity II system with RI and UV detectors (λ = 309 and 360 nm) for SEC analysis. The system included a PLGel 3 μm (50 × 7.5 mm) guard column and two PLGel 5 μm (300 × 7.5 mm) mixed-D columns. The eluent was DMF with 5 mM NH_4_BF_4_. Samples were prepared with a concentration of 10 mg mL^-1^ and filtered using 0.2 µm PTFE filters before auto-sampler injections. Calibration for molecular weight (*M*_w_) and molecular weight distributions (*Đ*_M_ = *M*_w_/*M*_n_) was done against poly (methyl methacrylate) (PMMA) standards, and analysis was conducted using Agilent SEC software.

**Transmission Electron Microscopy (TEM)**

We used dry-state transmission electron microscopy (TEM) with a JEOL 1400 Bio microscope at 80 kV acceleration voltage. After making the 0.10% w/w aqueous copolymer dispersion, we placed a 10.0 µL droplet on graphene oxide-coated copper grids for 1 minute, followed by careful blotting with filter paper to remove excess solution. Subsequently, the grid was stained with a 1 wt. % uranyl acetate (UA) solution for 30 seconds and then carefully blotted to remove excess stain before drying for microscopic analysis. To minimize any change in morphology before TEM analysis, 0.5 mg gel was incubated at different temperatures and then added 200 µl crosslinker stock solution [O, O´-1,3-propanediylbishydroxylamine dihydrochloride (O-alkyl hydroxylamine crosslinker), 1mg/ ml]. This was then diluted with 1800 µl H_2_O after equilibration for 20 minutes.^[2]^

**Rheology**

All rheological characterizations were performed on an Anton Parr MCR 302 rheometer fitted with a parallel plate PP08 (diameter of 8 mm). Rheology for the initial viscosity was conducted using a constant percentage strain of 1.0 % and a constant shear rate of 1.0 1/s, the initial data was recorded. Rheology for the modulus was conducted using a constant percentage strain of 1.0 % and a constant angular frequency of 0.1 rad/ s. Before the temperature-dependent test, the aqueous PHEAm_49_-*b*-(PHEAm_36_-*co*-PDAAm_115_) copolymer dispersion was equilibrated at every temperature for 5 min.

**Adhesion tests**

The adhesion strength of the hydrogels was evaluated using an Instron 5567 Universal Testing Machine at 25 °C, equipped with a 50 N load cell. Hydrogel samples of uniform weight (0.15 g) were placed on an aluminum plate substrate and evenly spread, followed by pressing with another aluminum plate (Figure S16). A preload of 10 N was applied for 10 seconds, after which the plates were separated at a rate of 1 mm/s in tensile mode, generating a force–distance curve. The adhesion strength was calculated by dividing the maximum force by the overlap adhesive area of the substrate.

To assess the temperature-dependent adhesion properties of the hydrogels, experiments were conducted at 1 °C, 25 °C, 35 °C, and 70 °C. The hydrogels were equilibrated at the respective temperatures for 5 hours to ensure complete morphological transitions. Then, adhesion testing started immediately after removing the samples from the fridge or oven. For reusability assessments, the same procedure was employed, with multiple testing cycles conducted to evaluate adhesion performance across repeated uses. For the cyclic tests, the attachment–detachment measurements were conducted following the same procedure, with an equilibration time of 10 minutes for each cycle. For long-term cycling tests, the equilibration time was reduced to 5 minutes per cycle.

For the adhesion test on AFM, imaging and analysis were conducted using a JPK NanoWizard 4 system operated in Quantitative Imaging (QI) mode. The AFM probes (PPP-NCHAuD, NANOSENSORS™) had resonance frequencies in the range of 204–497 kHz and force constants between 1–20 nN. Data acquisition and analysis were performed using JPK Data Processing software in QI mode.For sample preparation, the hydrogel was applied onto a glass substrate and pressed under a 10 N preload for 10 minutes to remove excess material and ensure uniform surface contact. To quantify the adhesion force as a function of temperature, a force mapping study was carried out. In this mode, force–displacement curves were recorded at 16,000 randomly distributed points across the hydrogel surface. The adhesion force at each point was determined from the retraction curve as the difference between the baseline and the minimum force value. All measurements were conducted within a 100 µm² (10 µm × 10 µm) scanning area, with acquisition frequencies ranging from 0.1 to 1.0 Hz.

**Synthesis and Characterization**

**Polymerization of Macro-CTA (PHEAm_49_)**

For the synthesis of the PHEAm_50_, 2-(butylthiocarbonothioylthio) propanoic acid (PABTC, 42.91 mg, 0.180 mmol), HEAm (1036.17 mg, 9.00 mmol), ACVA (5.05 mg, 0.018 mmol) and 1,4-dioxane: water 1:1 (2.067 mL) was all weighed into a vial and sealed with a rubber septum. The solution was mixed thoroughly in a sonic bath for 3 mins and then deoxygenated by bubbling N_2_ for 10 min. The vial was then placed in an oil bath set at 70 °C for 5 h. Reaction aliquots to determine conversion via ^1^H NMR spectroscopy was taken using a degassed syringe. After the polymerization, the mixture was cooled and opened to air. The crude polymer was precipitated three times in 1,4-dioxane to remove any residual monomer and dried in *vacuo*. The product was isolated as a yellow solid powder.

| **Polymer** | **wt (%)** | **DP** | **Conv. (%)** | ***M*_n_, _NMR_ (g mol^-1^)** | ***M*_n_, _SEC_ (kg mol^-1^)** | ***Ð* _M_** |
| --- | --- | --- | --- | --- | --- | --- |
| **PHEAm_49_** | 40 | 50 | 97 | 5879 | 4.11 | 1.07 |
| **PDMA_49_** | 40 | 50 | 99 | 5095 | 3.07 | 1.07 |

**Table S1.** The summary of polymerization of Macro-CTA (PHEAm_49_), with actual and target DPs, conversions, molar mass, and weight ratios after chain extension.

^1^H NMR spectroscopy studies indicated that > 99% HEAm conversion was achieved within 4 h, as determined by comparing the HEAm -NH- signals at 5.7–5.5 ppm at different reaction time. DMF SEC analysis indicated a *M*_n_ of 5879 g mol^−1^ and an *M*_w_/*M*_n_ of 1.07, respectively. The PDMA_50_ polymer was synthesized and analysed by ^1^H NMR in the same way, with a *M*_n_ of 5095 g mol^−1^ and an *M*_w_/*M*_n_ of 1.07.

**General PISA protocol**

Macro-CTA (PHEAm_49_, 137.18 mg, 0.023mmol, 1.00 eq.), diacetone acrylamide (DAAm) (444.20 mg, 2.63 mmol), HEAm (100.74 mg, 0.88 mmol), target DP = 150. ACVA initiators (0.267 mg, 0.954 µmol, 0.500 eq.) were dissolved in H_2_O (1 g, corresponding to 10% w/v total solid content at full conversion). The solution was mixed thoroughly and deoxygenated by bubbling nitrogen for 10 min. The vial was then placed in an oil bath set at 70 °C for 2 h. The polymerizations were quenched by removing the vial from the oil bath and opening it to air.

**Table S2.** The summary of polymerization of PHEAm_49_-*b*-(PHEAm_36_-*co*-PDAAm_115_) with actual and target DPs, conversions, molar mass, and weight ratios after chain extension.

| **Polymer** | **wt (%)** | **Target DP**  **(HEAm, DAAm)** | **Conv. HEAm (%)** | **Conv. DAAm (%)** | ***M*_n_, _NMR_ (g mol^-1^)** | ***M*_n_, _SEC_ (kg mol^-1^)** | ***Ð* _M_** |
| --- | --- | --- | --- | --- | --- | --- | --- |
| **PHEAm_49_-*b*-(PHEAm_12_-*co*-PDAAm_39_)** | 42.8 | 12, 39 | 97 | 98 | 13860 | 10.4 | 1.12 |
| **PHEAm_49_-*b*-(PHEAm_24_-*co*-PDAAm_74_)** | 42.8 | 100 | 97 | 99 | 21164 | 17.9 | 1.14 |
| **PHEAm_49_-*b*-(PHEAm_36_-*co*-PDAAm_115_)** | 42.8 | 150 | 97 | 99 | 29483 | 24.5 | 1.12 |
| **PHEAm_49_-*b*-(PHEAm_47_-*co*-PDAAm_151_)** | 42.8 | 200 | 96 | 99 | 36842 | 26.1 | 1.22 |
| **PHEAm_49_-*b*-(PHEAm_70_-*co*-PDAAm_222_)** | 42.8 | 300 | 94 | 98 | 51504 | 49.0 | 1.10 |
| **PDMA_49_-*b*-(PHEAm_38_-*co*-PDAAm_114_)** | 42.8 | 150 | 99 | 99 | 28761 | 18.9 | 1.10 |
| **PHEAm_49_-*b*-(PHEAm_40_-*co*-PDAAm_114_)** | 29.2 | 150 | 95 | 98 | 29775 | 23.5 | 1.18 |
| **PHEAm_49_-*b*-(PHEAm_35_-*co*-PDAAm_110_)** | 34.3 | 150 | 97 | 99 | 28522 | 23.0 | 1.18 |
| **PHEAm_49_-*b*-(PHEAm_36_-*co*-PDAAm_115_)** | 38.0 | 150 | 96 | 99 | 29483 | 18.8 | 1.22 |

**Figure S1.** SEC results for different Macro-CTA: PHEAm_49_ and PDMA_49_ after chain extension polymerization.


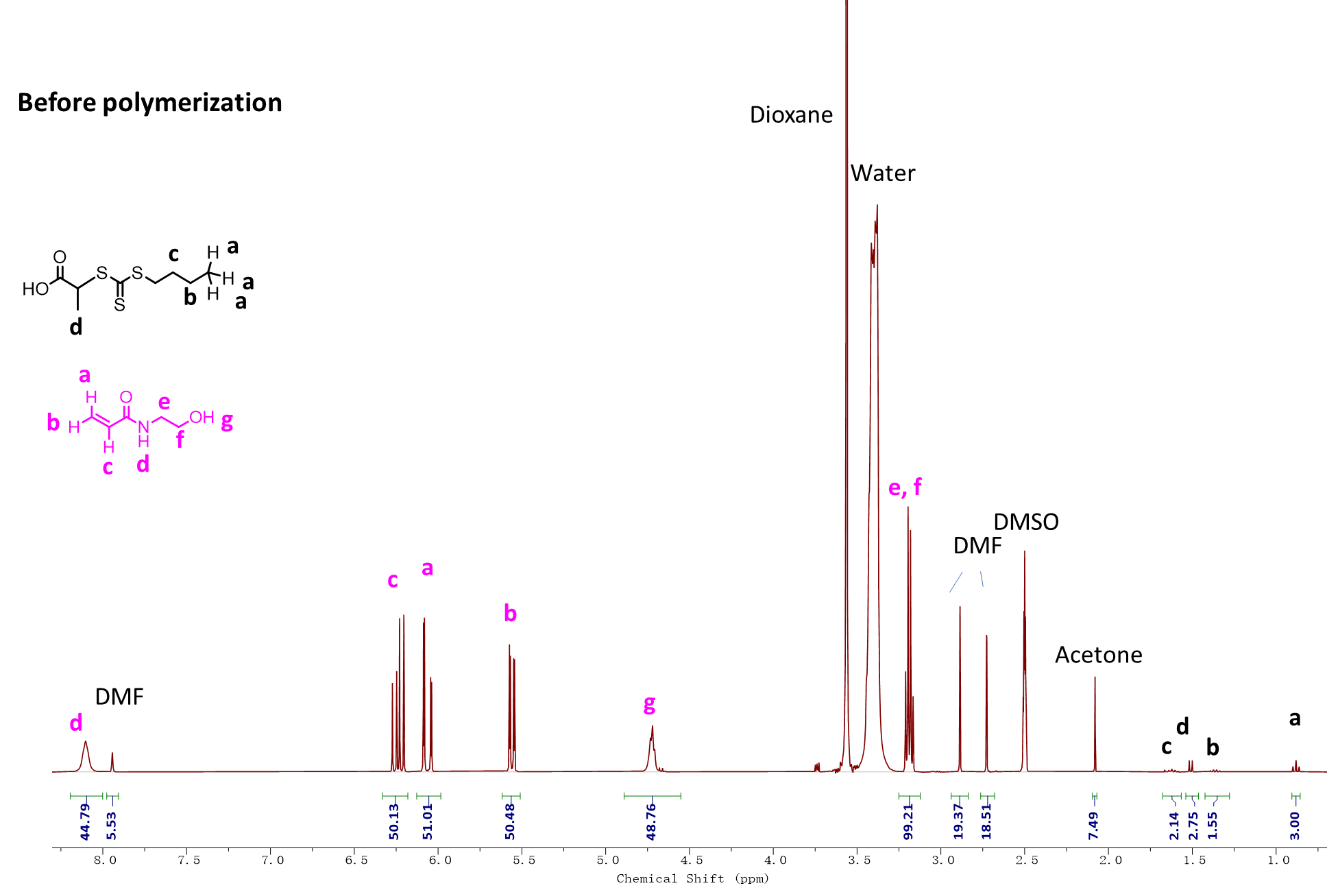


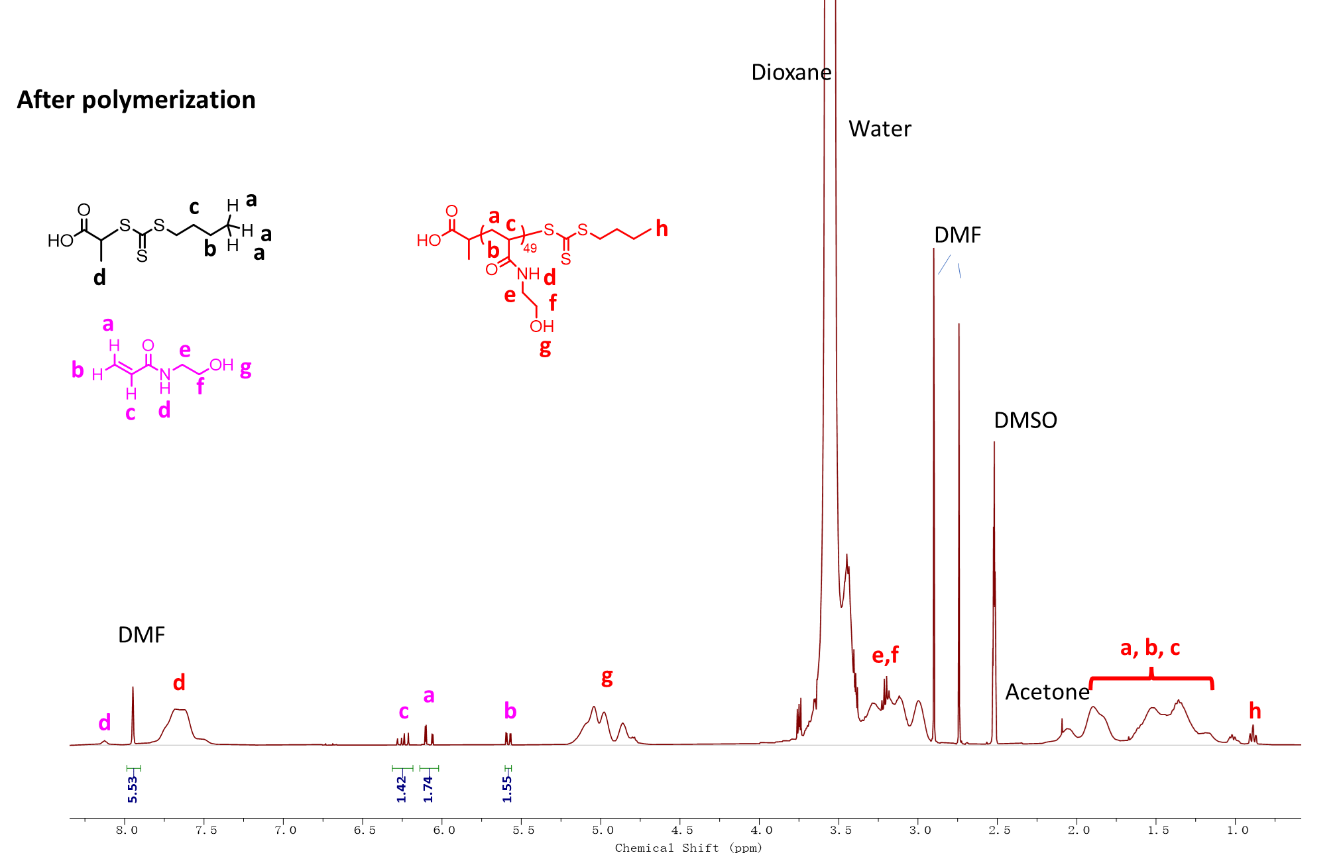


**Figure S2.** ^1^H NMR spectrum of PHEAm_49_ in deuterated DMSO (400 MHz) (42.8 wt%) before and after polymerization.


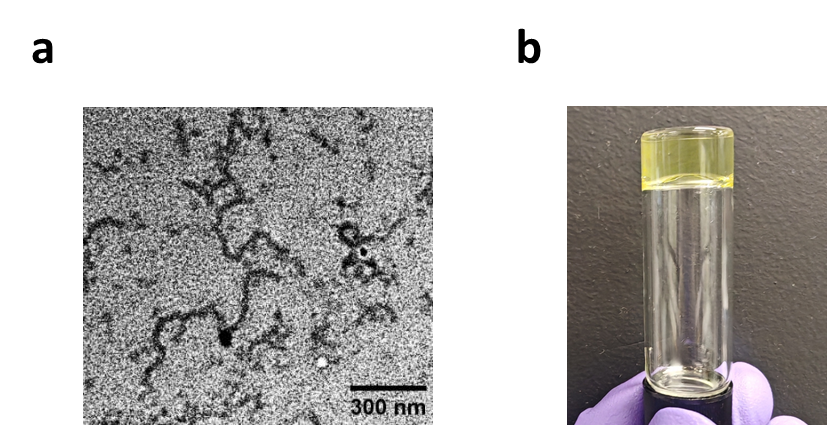


**Figure S3.** a) TEM image of cylindrical micelles (i.e. worms) for PHEAm_49_-*b*-(PHEAm_36_-*co*-PDAAm_115_) adhesive hydrogel (42.8 wt%, 25 °C); b) Picture of the freestanding worm-based hydrogel.


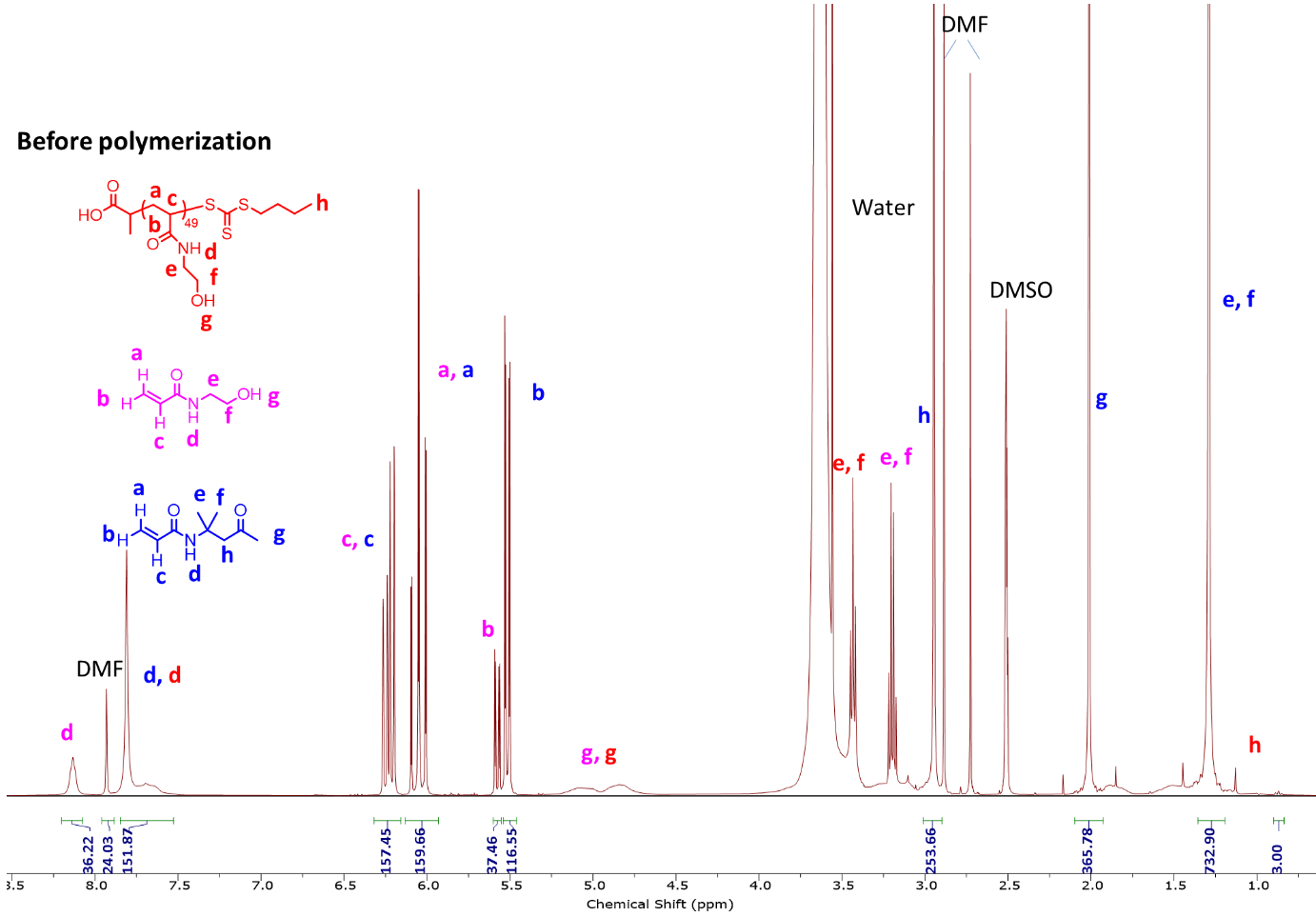


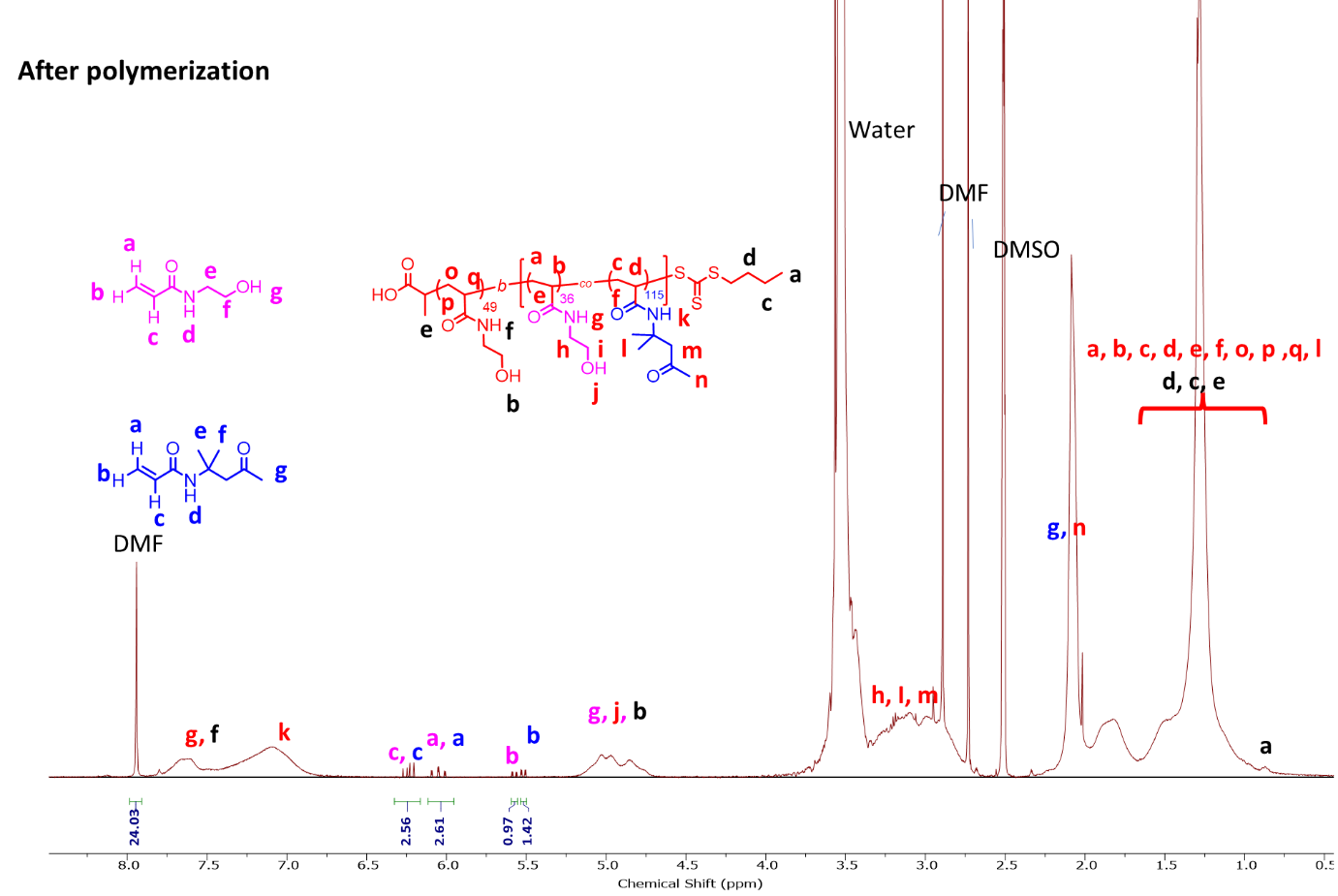


**Figure S4.** ^1^H NMR spectrum of PHEAm_49_-*b*-(PHEAm_36_-*co*-PDAAm_115_) (42.8 wt%) in deuterated DMSO (400 MHz) before and after polymerization.


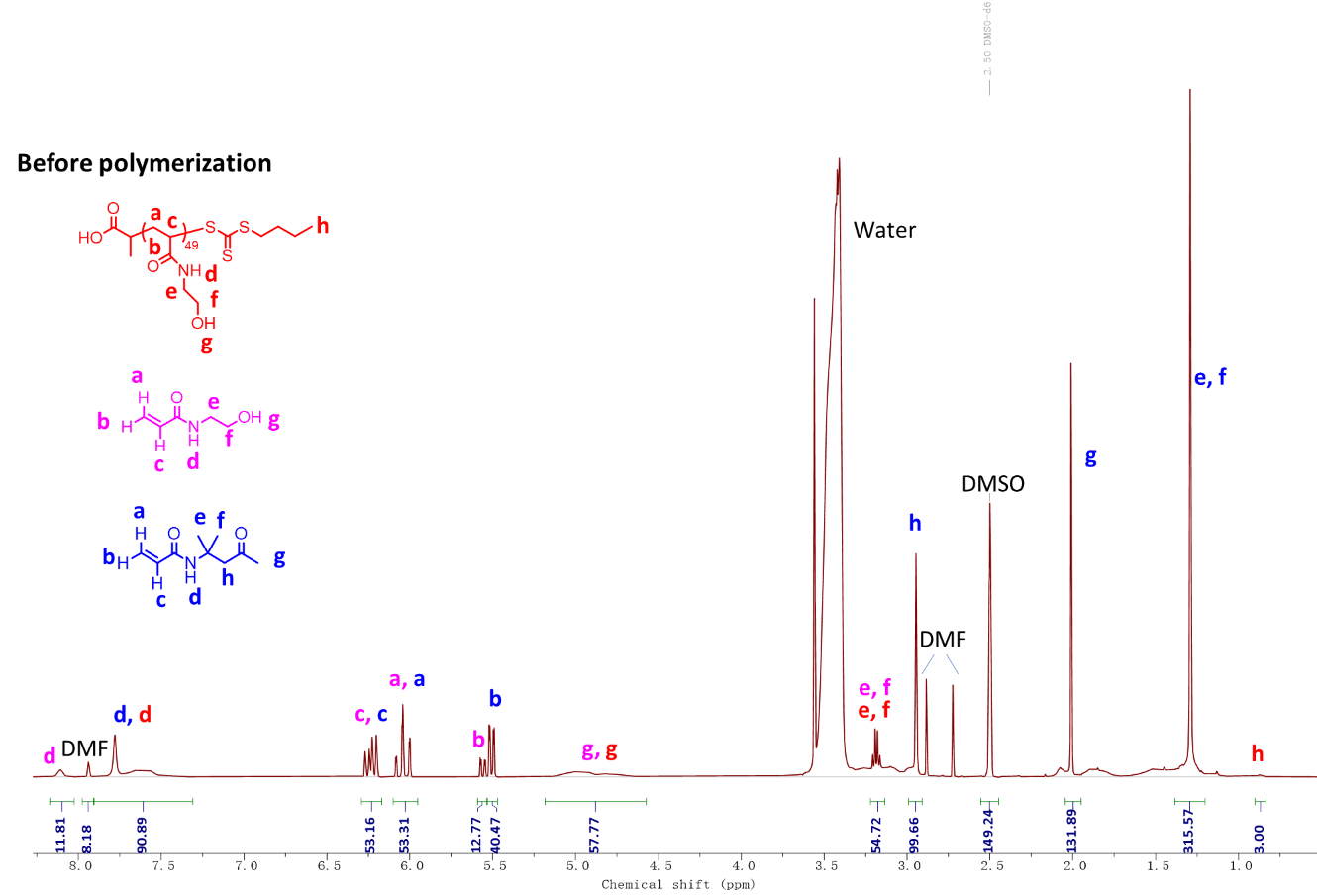


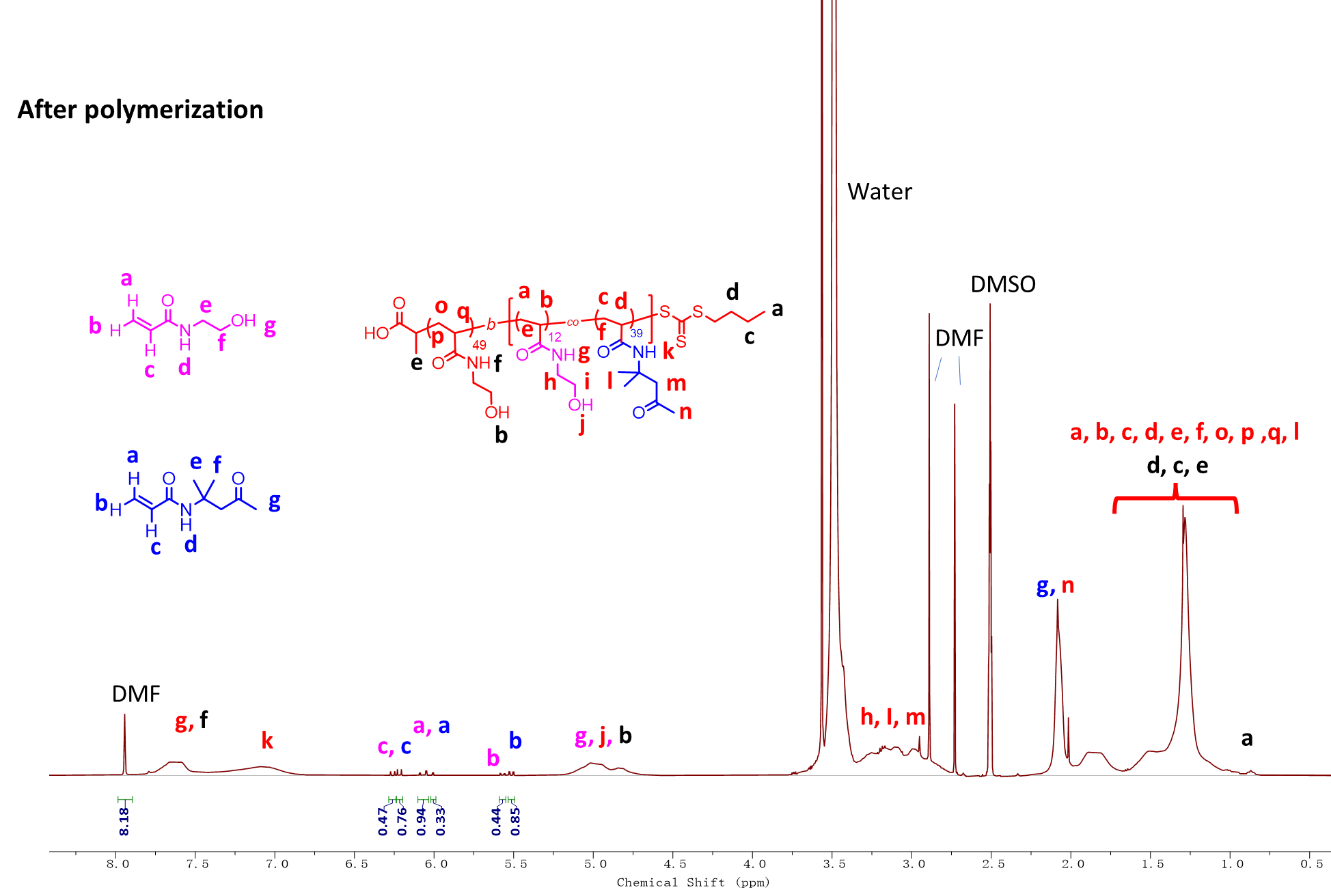


**Figure S5.** ^1^H NMR spectrum of PHEAm_49_-*b*-(PHEAm_12_-*co*-PDAAm_39_) (42.8 wt%) in deuterated DMSO (400 MHz) before and after polymerization.


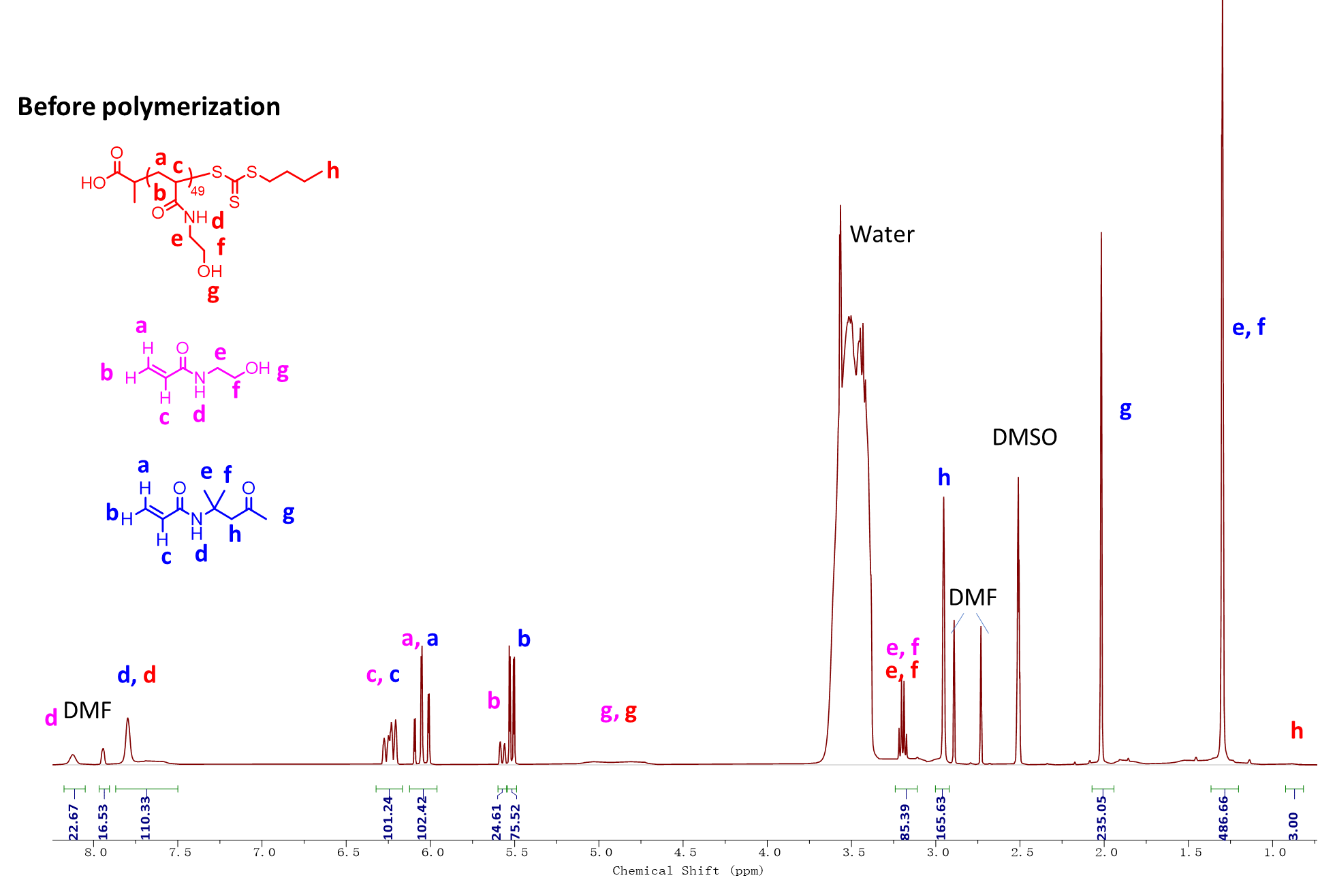


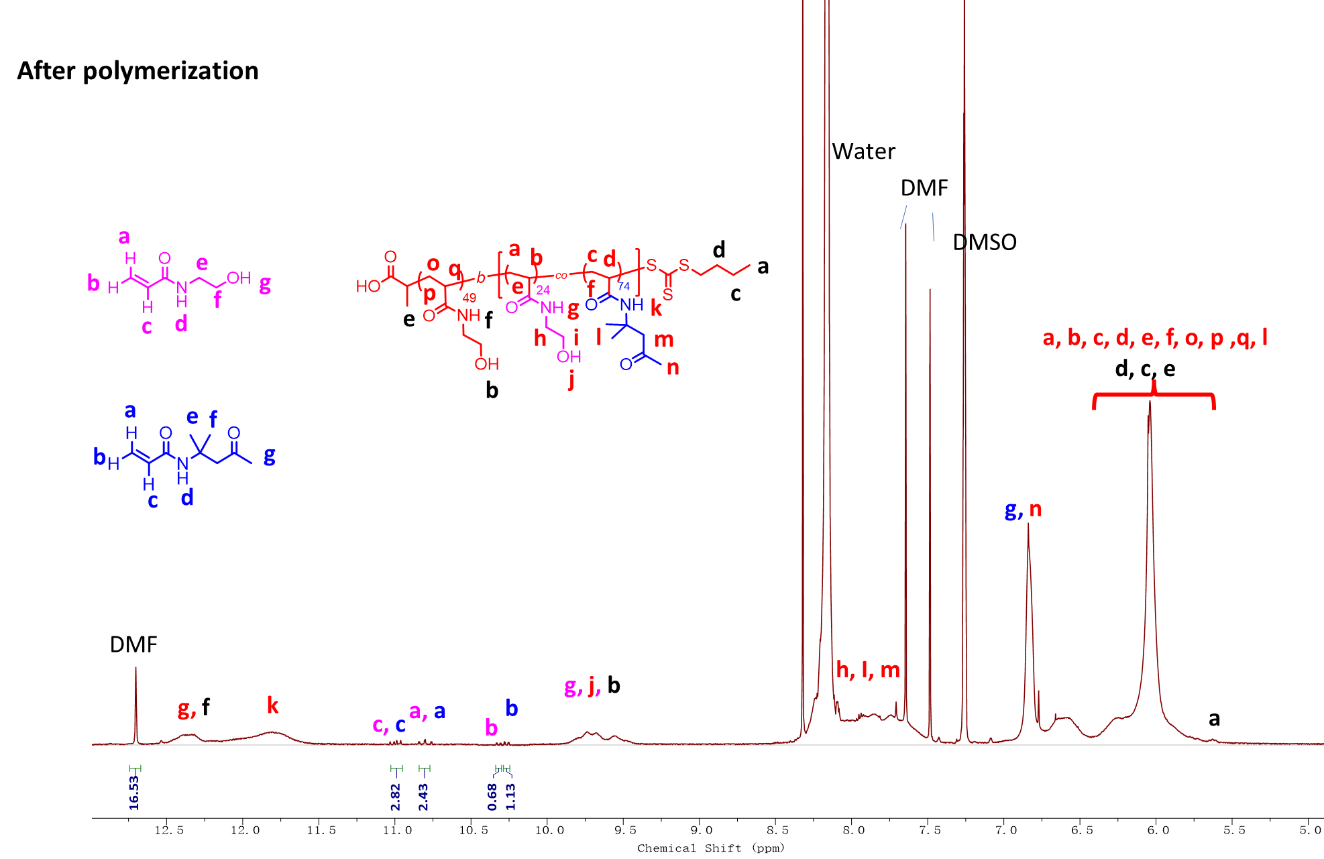


**Figure S6.** ^1^H NMR spectrum of PHEAm_49_-*b*-(PHEAm_24_-*co*-PDAAm_74_) (42.8 wt%) in deuterated DMSO (400 MHz) before and after polymerization.


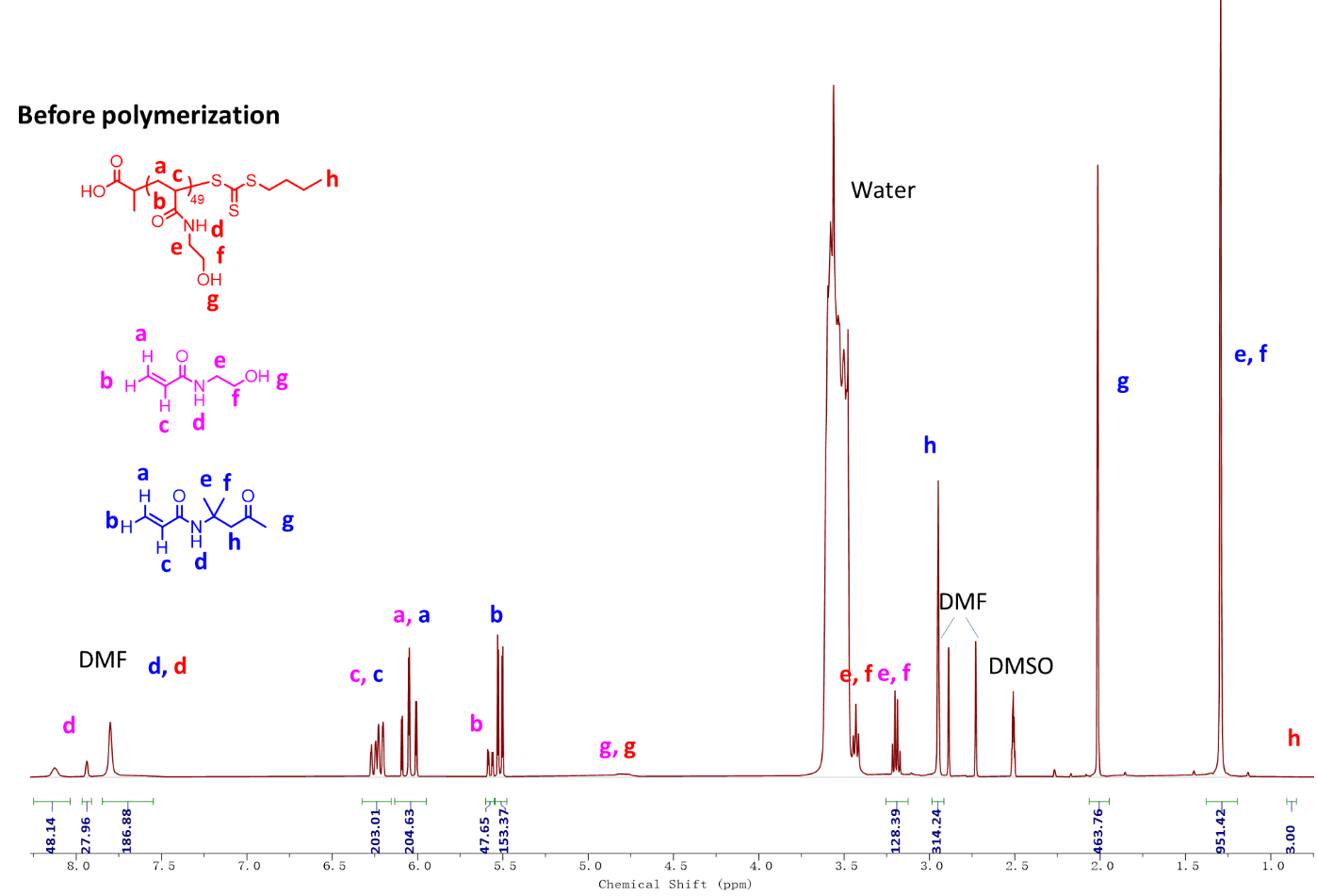


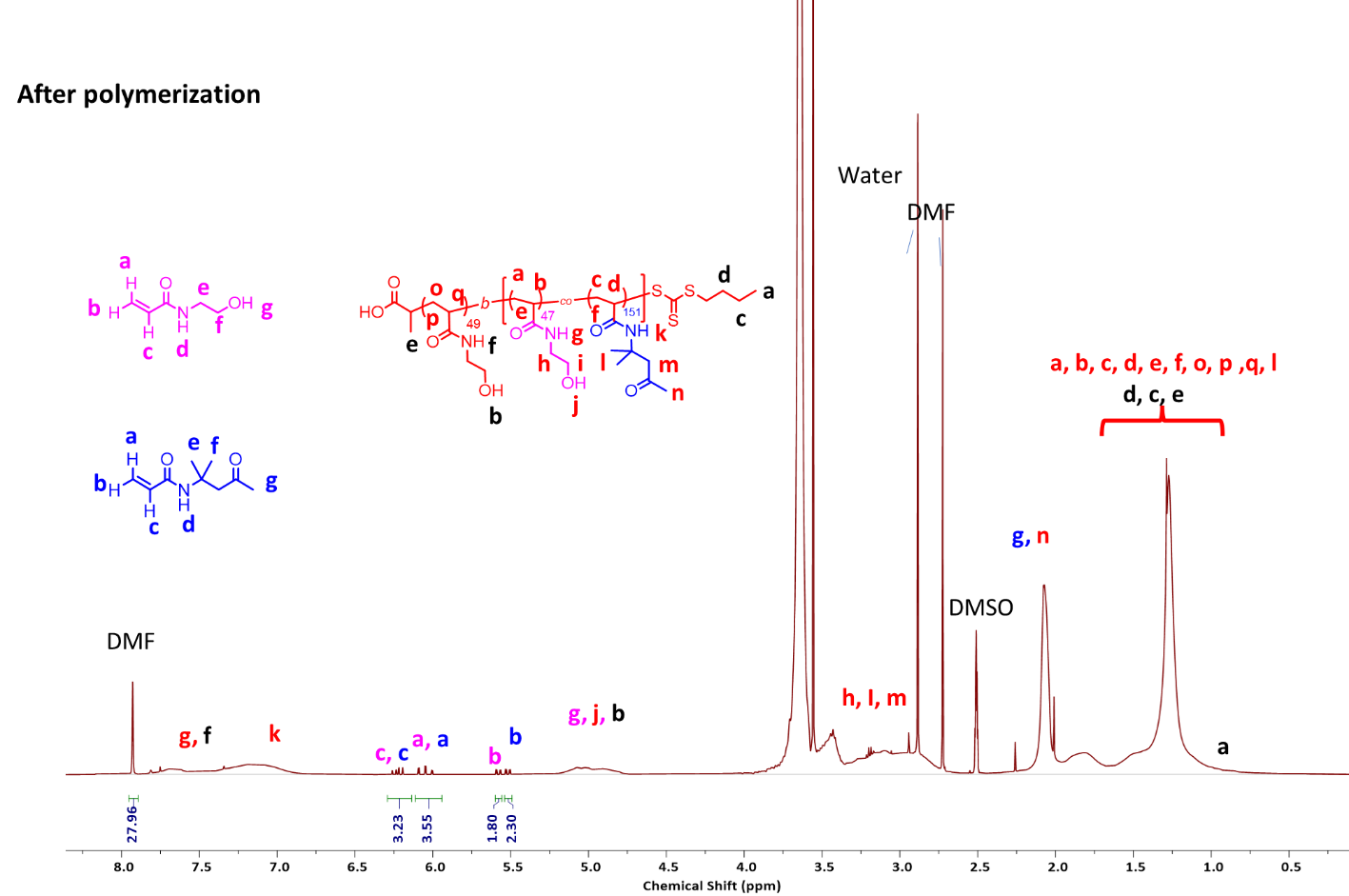


**Figure S7.** ^1^H NMR spectrum of PHEAm_49_-*b*-(PHEAm_47_*-co*-PDAAm_151_) (42.8 wt%) in deuterated DMSO (400 MHz) before and after polymerization.


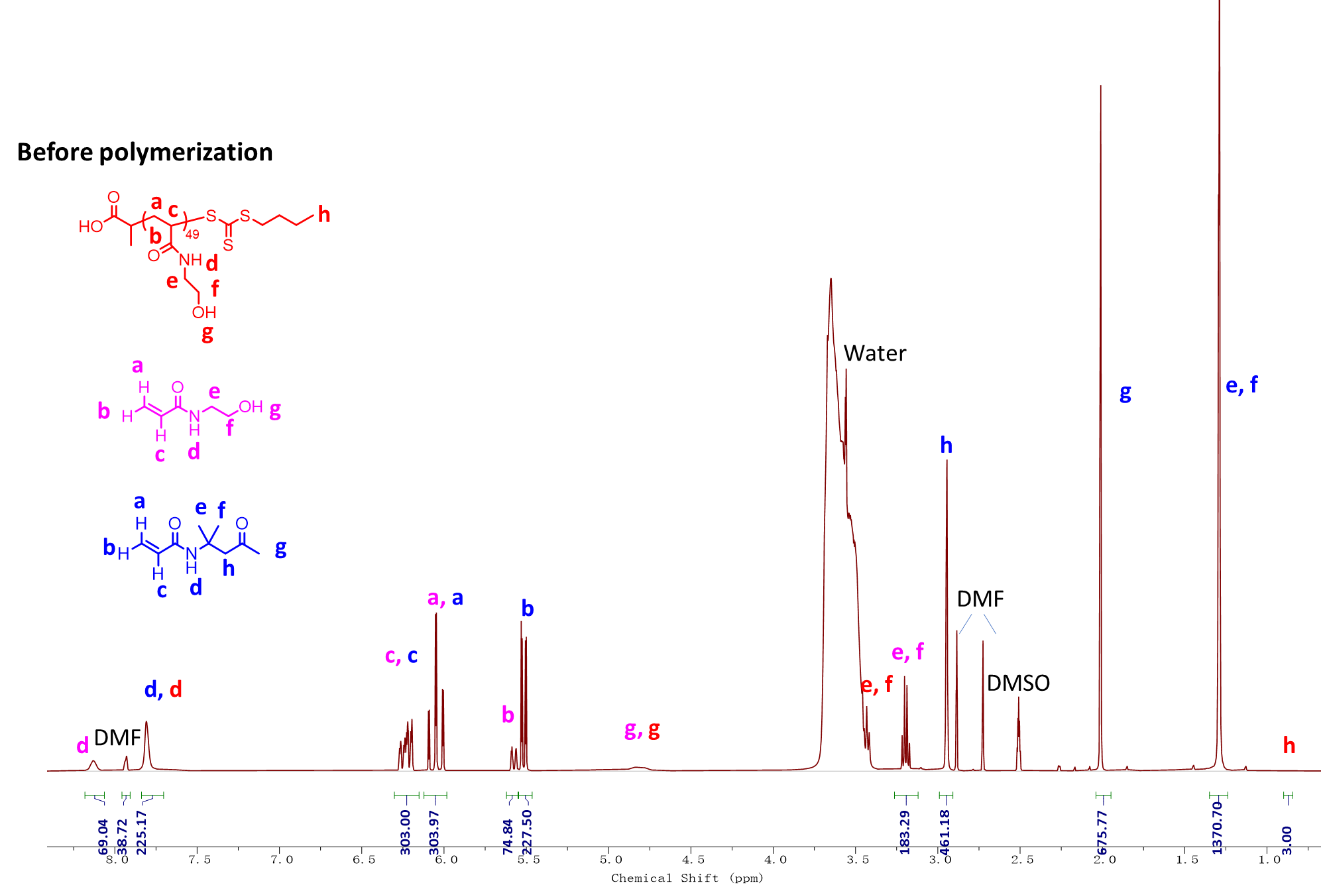


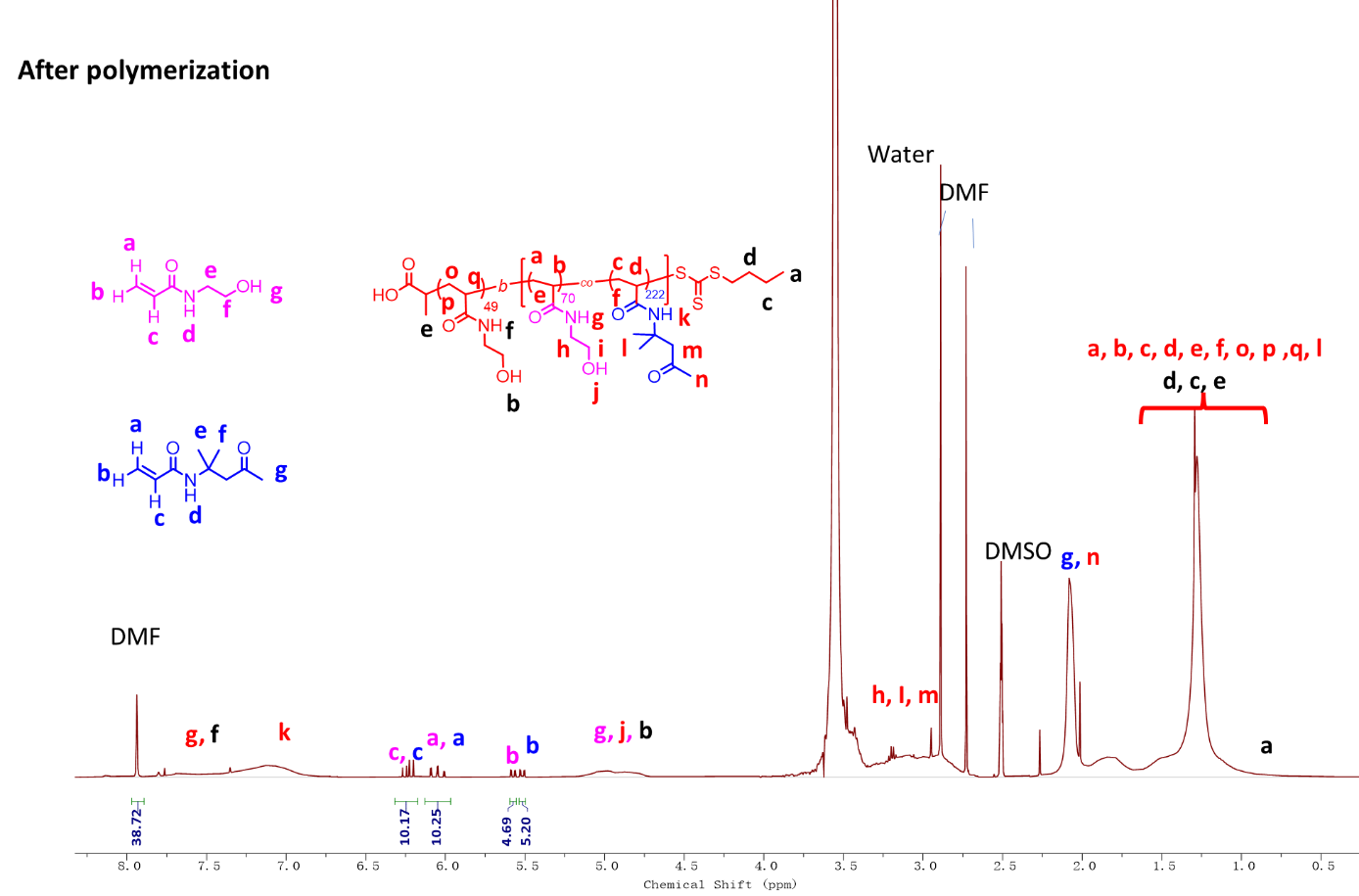


**Figure S8.** ^1^H NMR spectrum of PHEAm_49_-*b*-(PHEAm_70_-*co*-PDAAm_222_) (42.8 wt%) in deuterated DMSO (400 MHz) before and after polymerization.


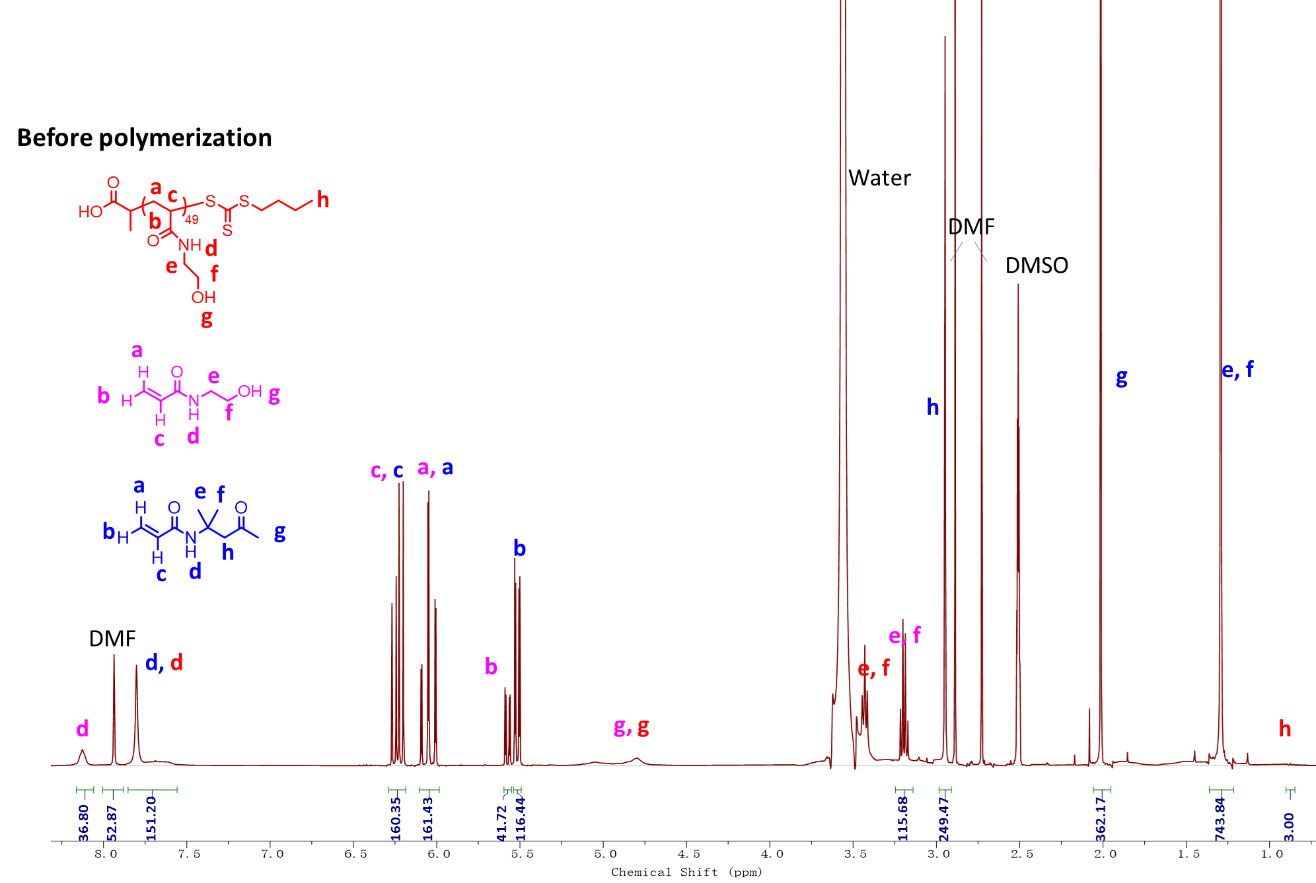


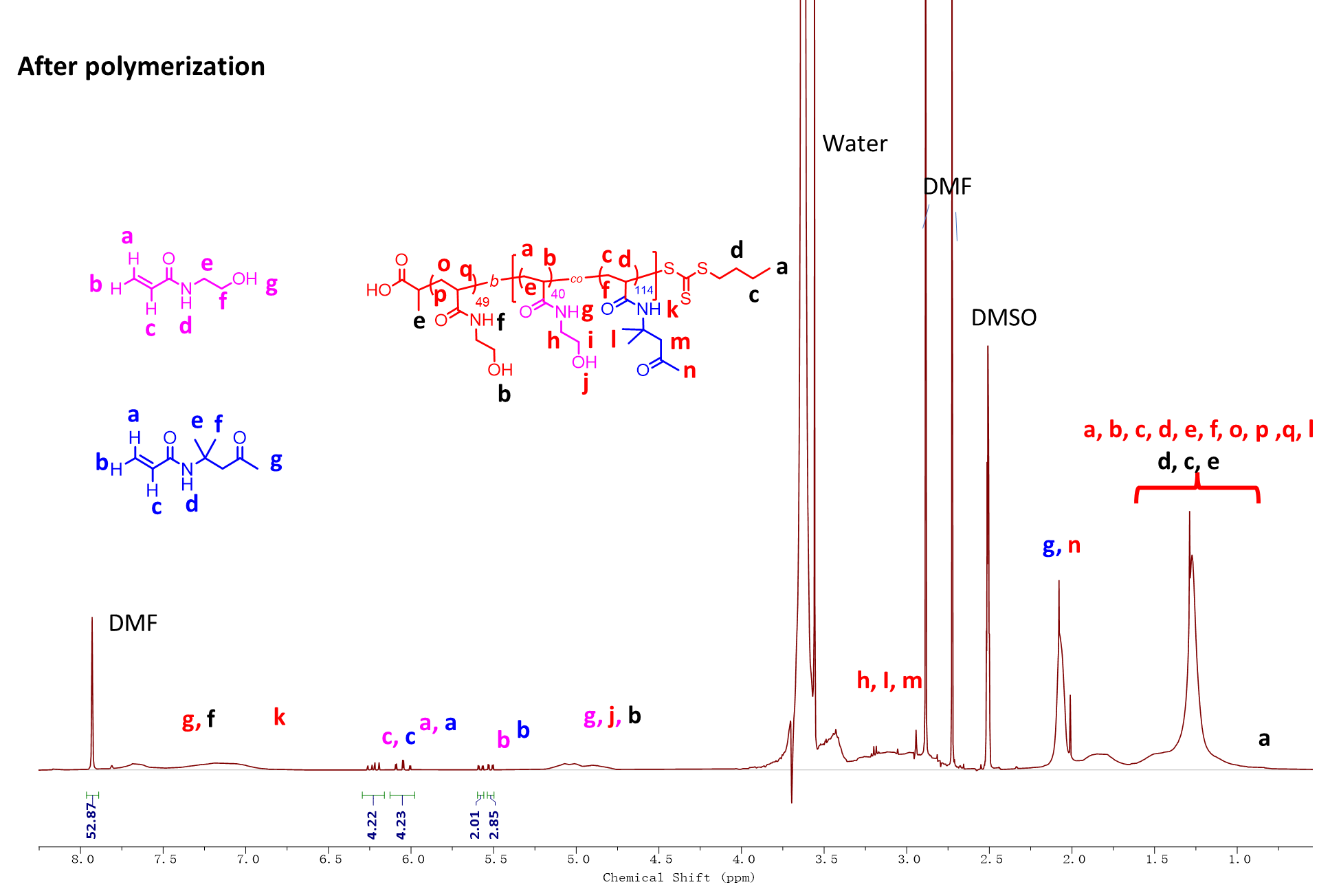


**Figure S9.** ^1^H NMR spectrum of HEAm_49_-*b*-(PHEAm_40_-*co*-PDAAm_114_) (29.2 wt%) in deuterated DMSO (400 MHz) before and after polymerization.


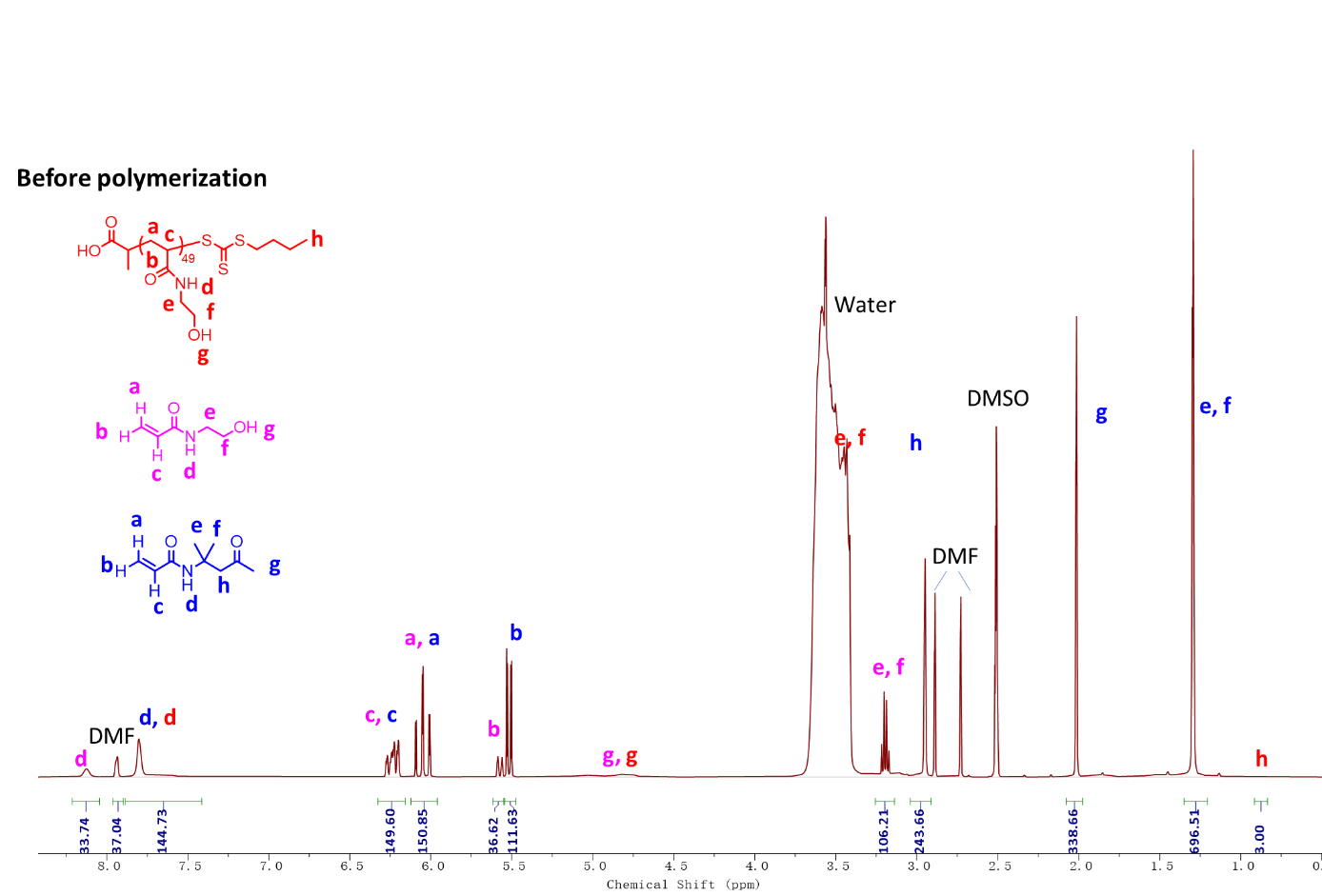


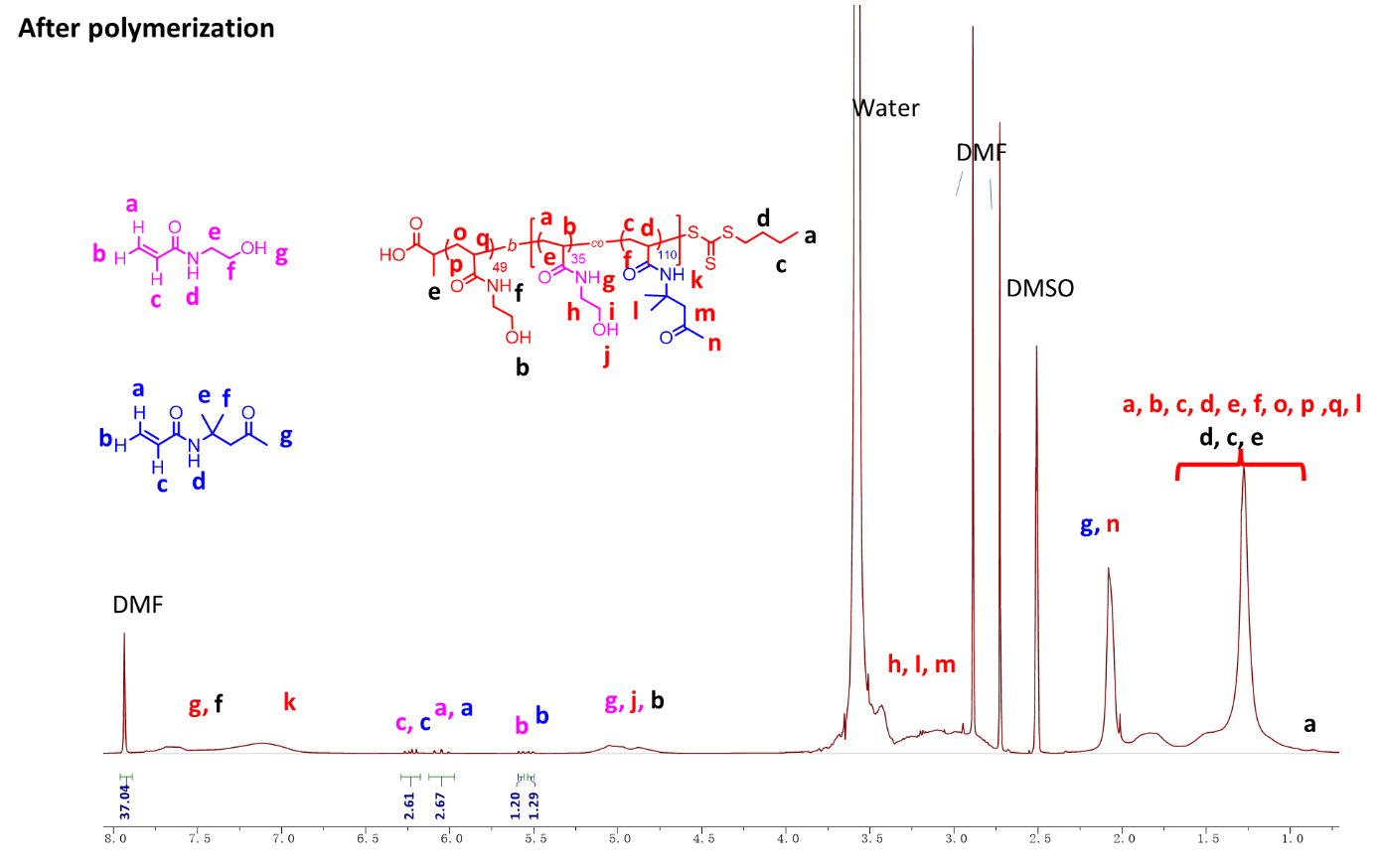


**Appendix S8.** ^1^H NMR spectrum of PHEAm_49_-*b*-(PHEAm_35_-*co*-PDAAm_110_) (34.3 wt%) in deuterated DMSO (400 MHz) before and after polymerization.


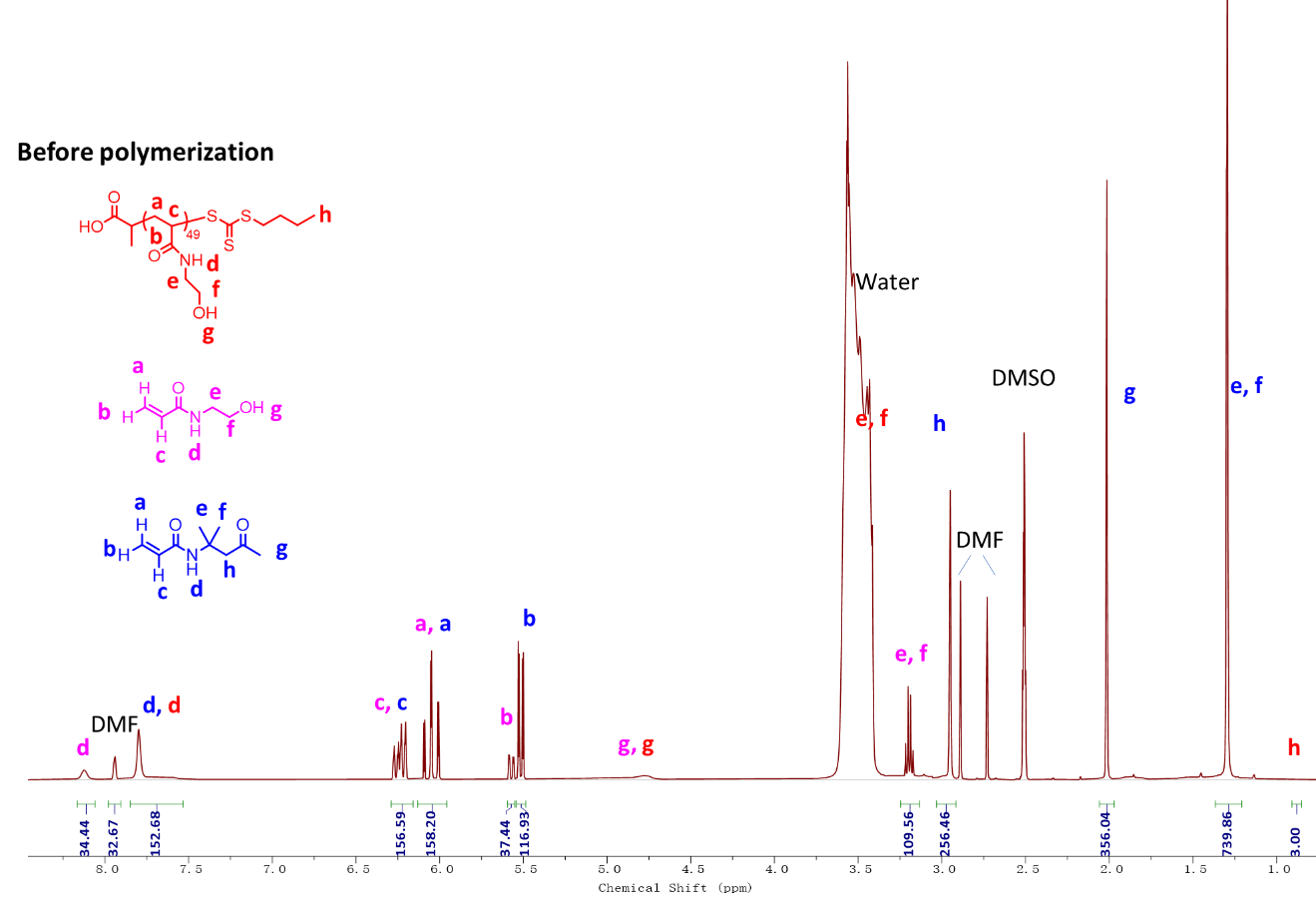


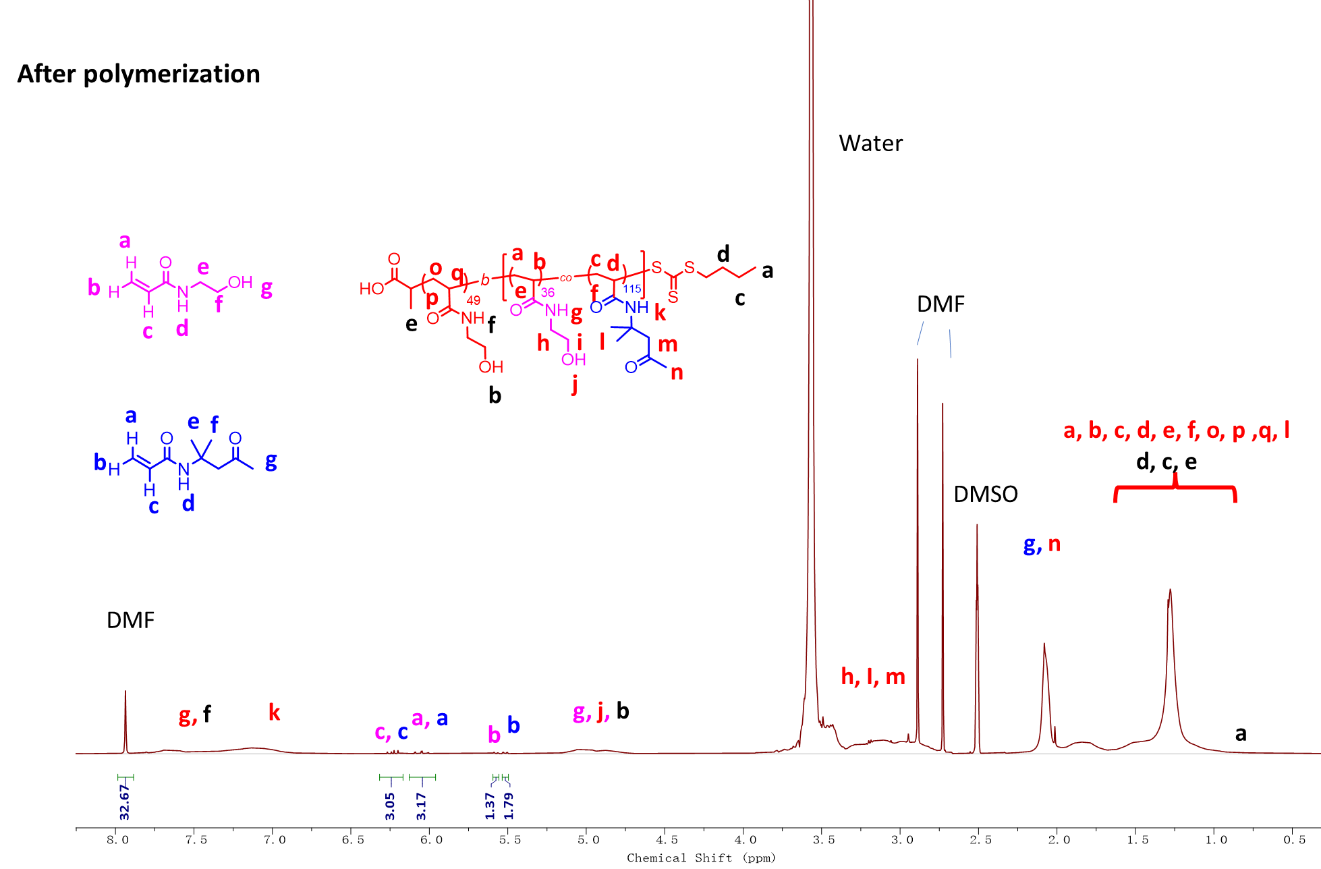


**Appendix S9.** ^1^H NMR spectrum of PHEAm_49_-*b*-(PHEAm_36_-*co*-PDAAm_115_) (38 wt%) in deuterated DMSO (400 MHz) before and after polymerization.

**Figure S10.** SEC results for different DPs after chain extension.

**Figure S11.** SEC results for different weight ratios after chain extension.


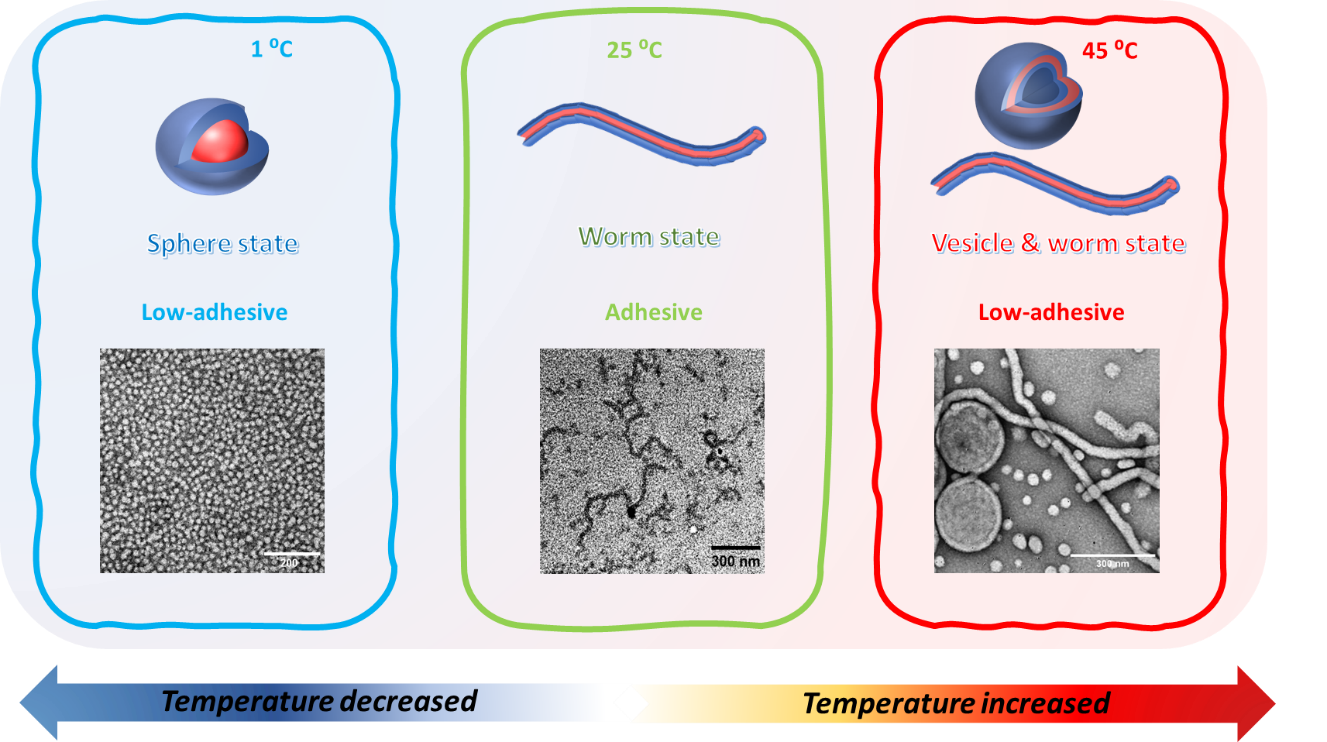


**Figure S12.** Schematic illustration of the transitions of bidirectional temperature-responsive morphologies triggered by the worm-based hydrogel, which can be selectively switched to a viscous liquid state when the morphologies change at different temperatures.


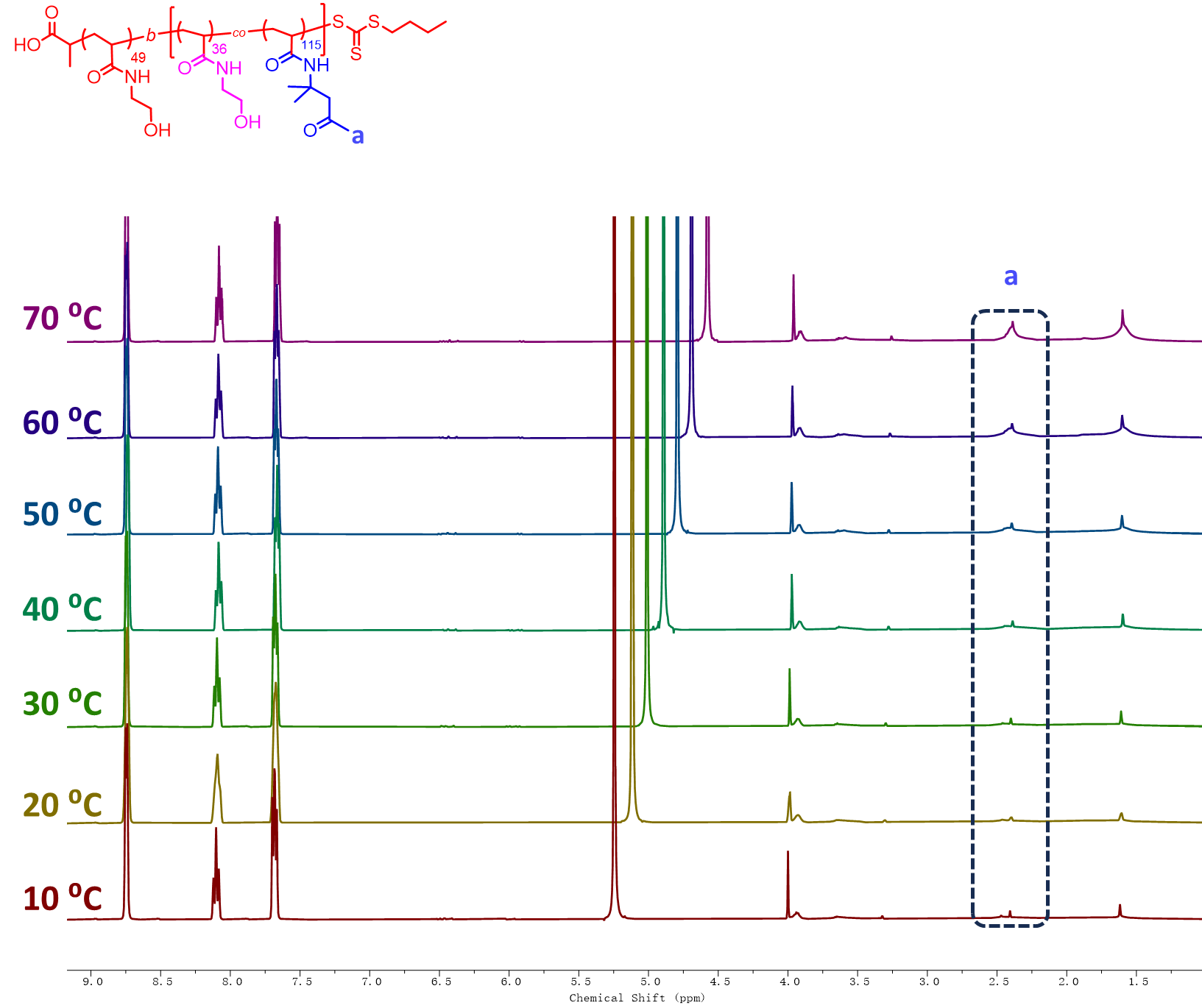


**Figure S13.** Variable temperature ^1^H NMR for PHEAm_49_-*b*-(PHEAm_36_-*co*-PDAAm_115_) (from 10⁰C - 70⁰C, 42.8 wt%), and normalised relative to the external standard pyridine.

**Figure S14.** Viscosity changes of PHEAm_49_-*b*-(PHEAm_36_-*co*-PDAAm_115_) adhesive hydrogel from 1 °C to 70 °C, every 5 °C after equilibrium for 10 mins.


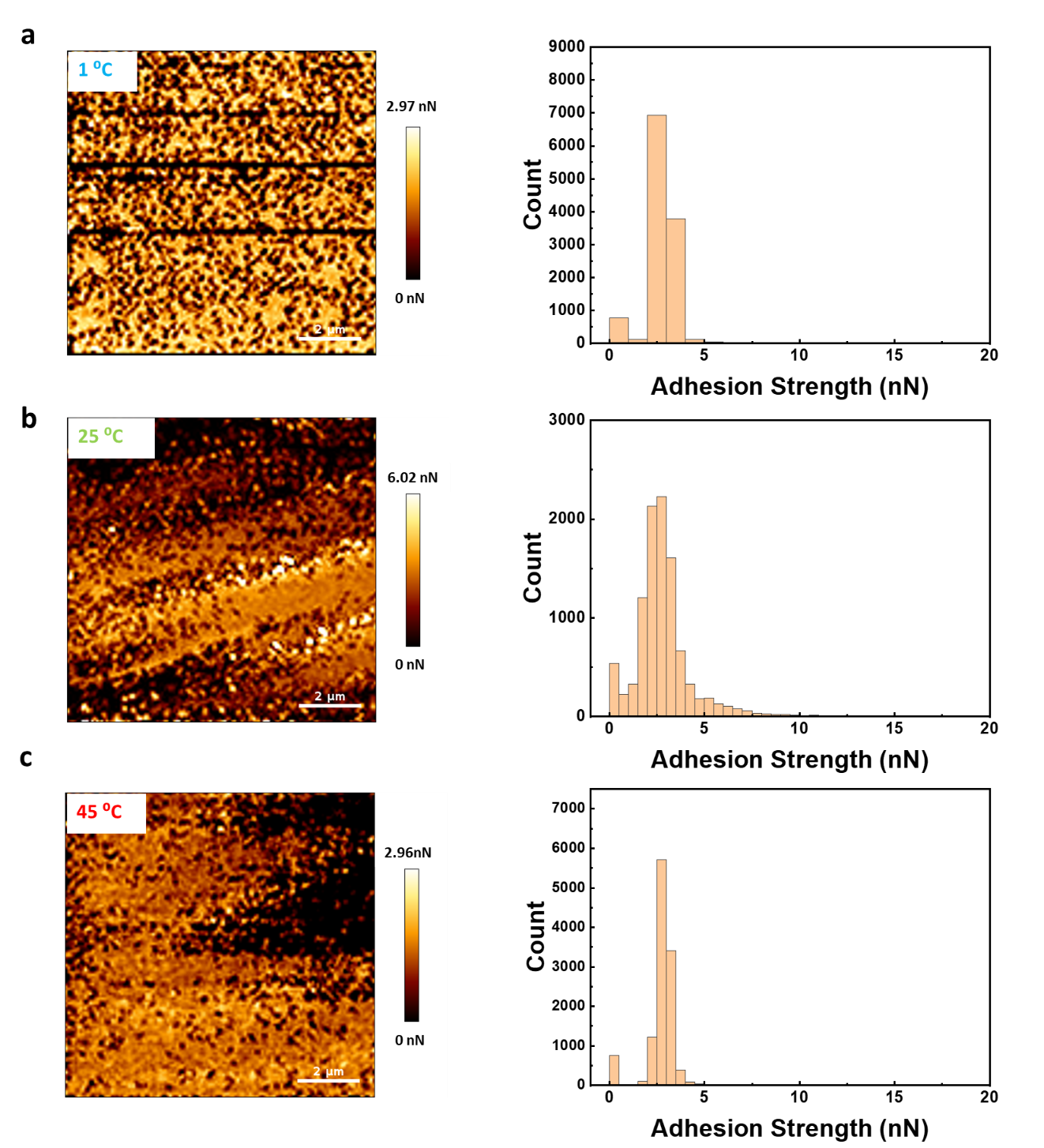


**Figure S15.** Relative adhesion strength map and corresponding histograms of PHEAm_49_-*b*-(PHEAm_36_-*co*-PDAAm_115_) measured at different temperatures: (a) 1 °C, (b) 25 °C, and (c) 45 °C.


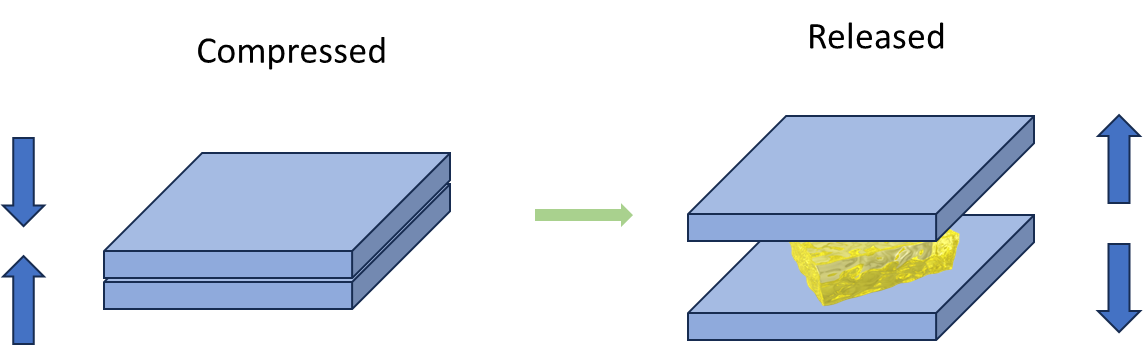


**Figure S16.** Schematic diagram for tensile adhesion test.


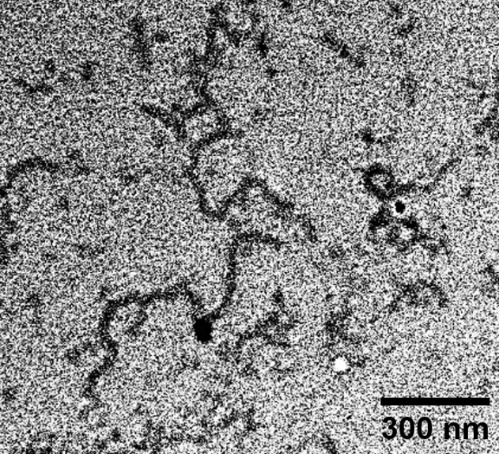

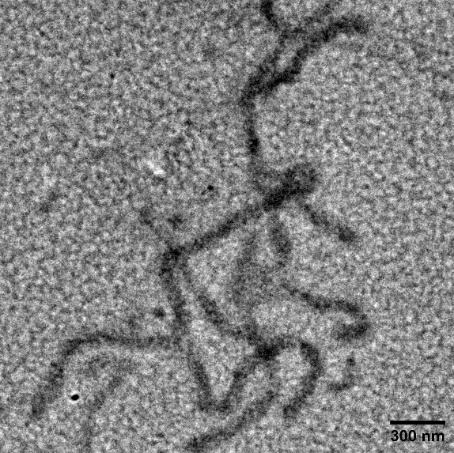


**Figure S17.** TEM results for worm-based hydrogel at different DPs, left side is DP150 (average thickness 24 nm) and the right is DP 300 (average thickness 75 nm).


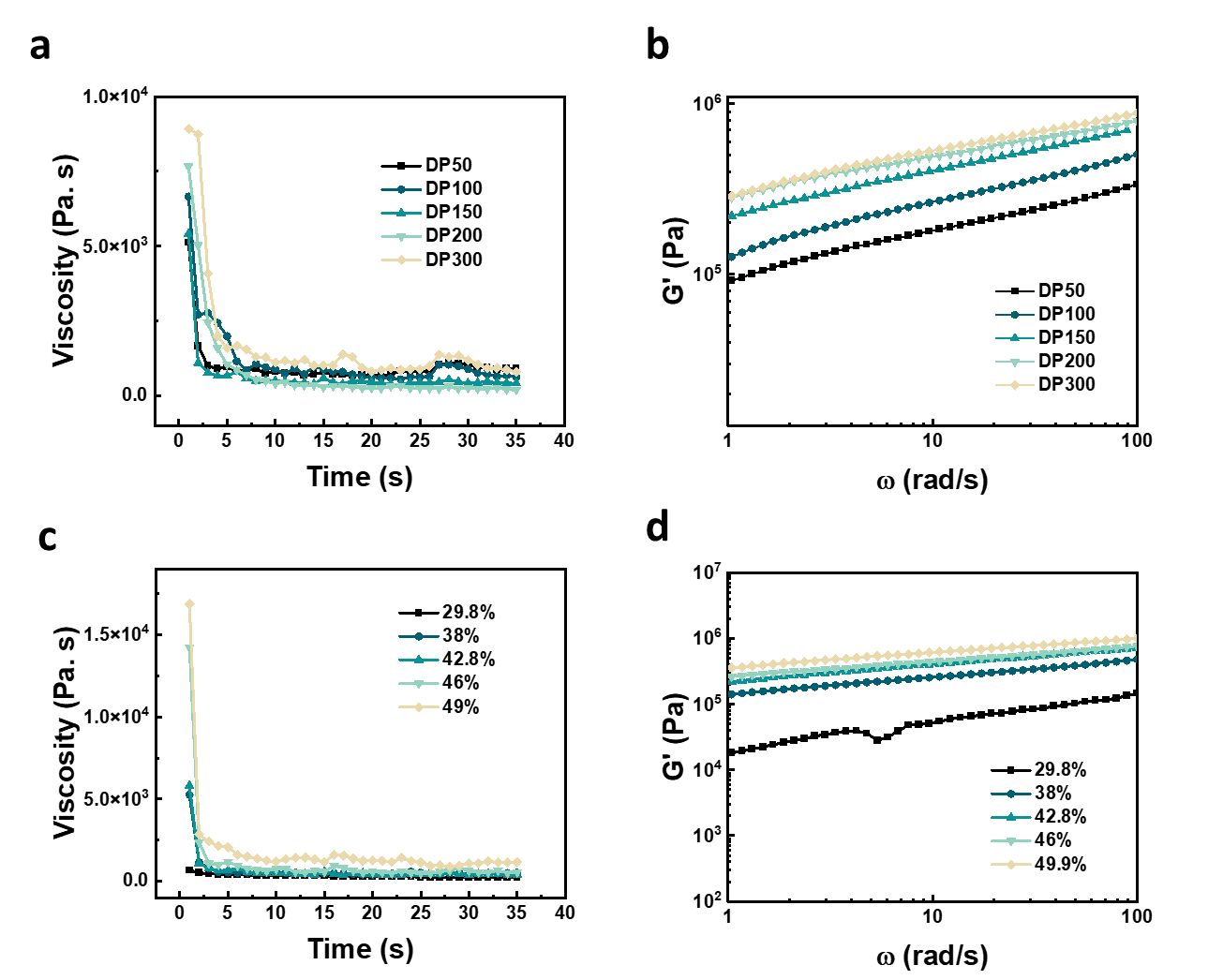


**Figure S18.** The viscosity and modulus at different DPs are shown in a) and b). The viscosity and modulus at different weight ratios are shown in c) and d).


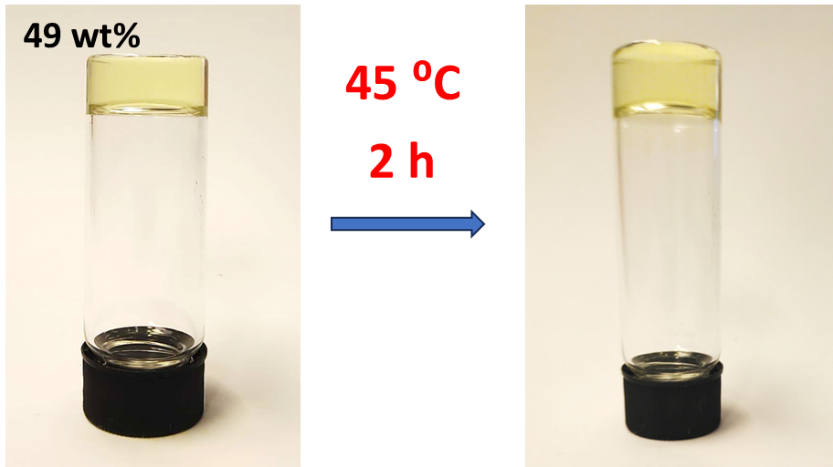


**Figure S19.** The high-temperature responsive phase transition for PHEAm_49_-*b*-(PHEAm_36_-*co*-PDAAm_115_) adhesive hydrogel was difficult to observed when the weight ratio exceeded 42.8%).


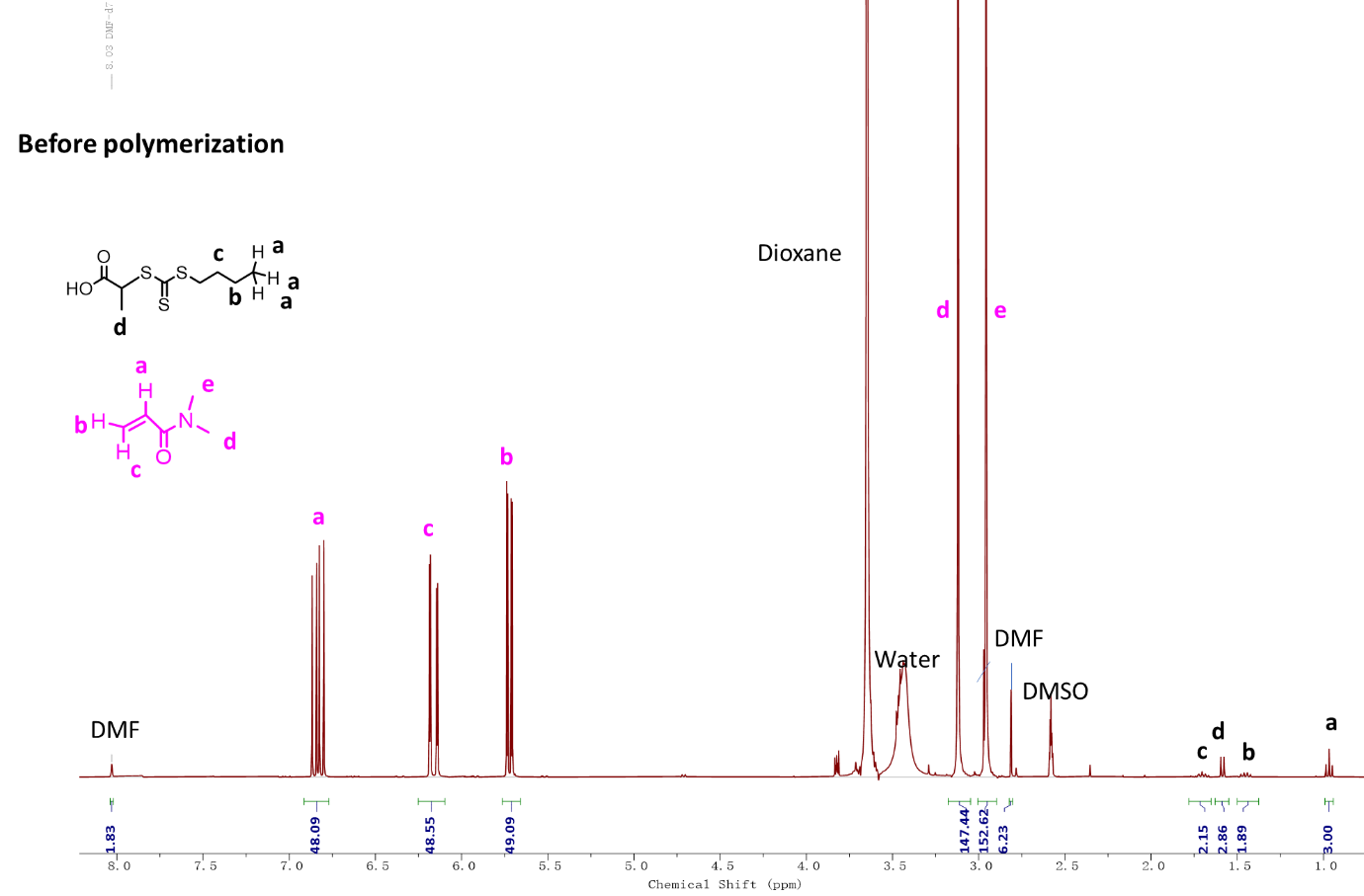


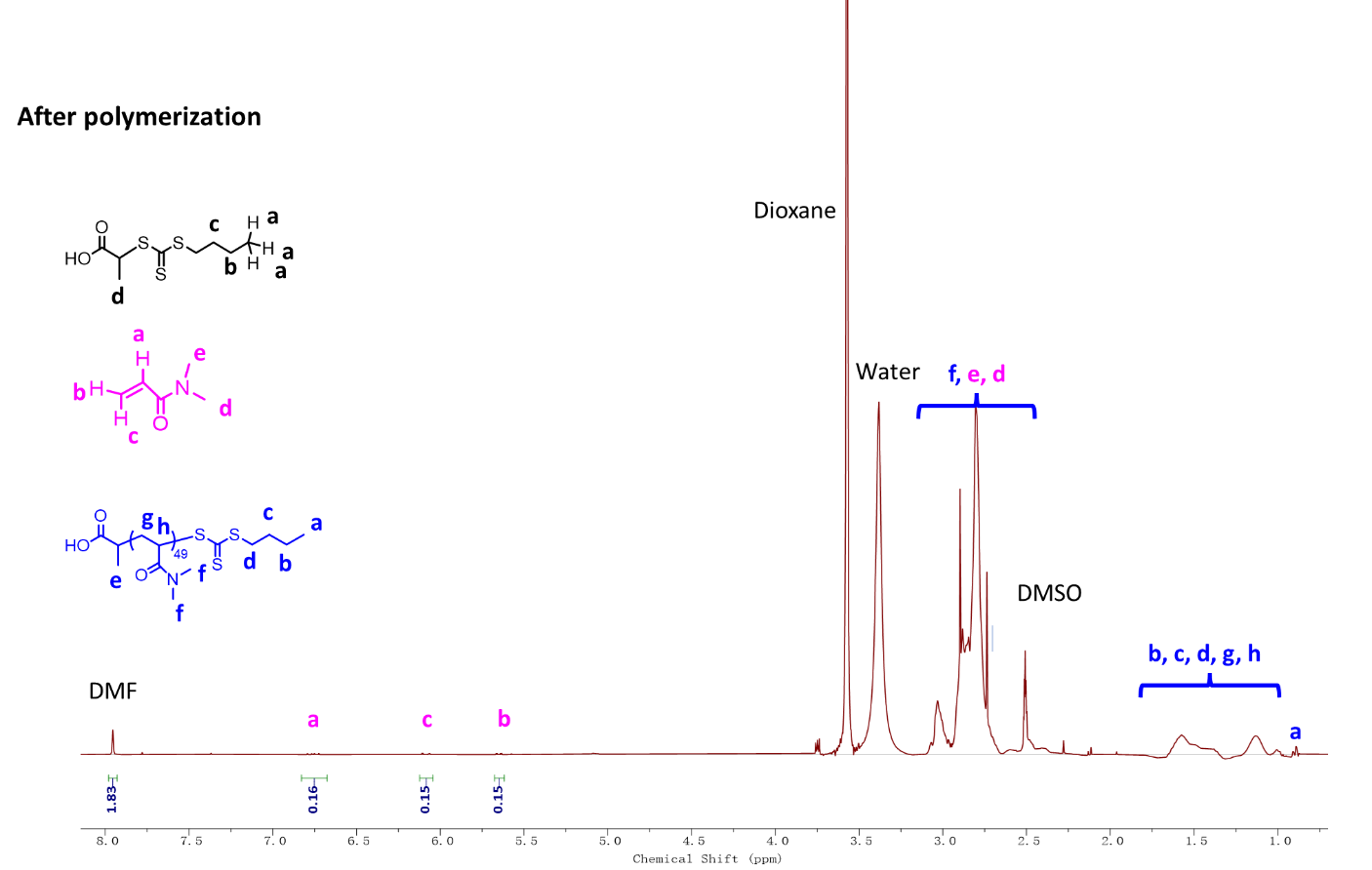


**Figure S20.** ^1^H NMR spectrum of PDMA_49_ in deuterated DMSO (400 MHz) (42.8 wt%) before and after polymerization.


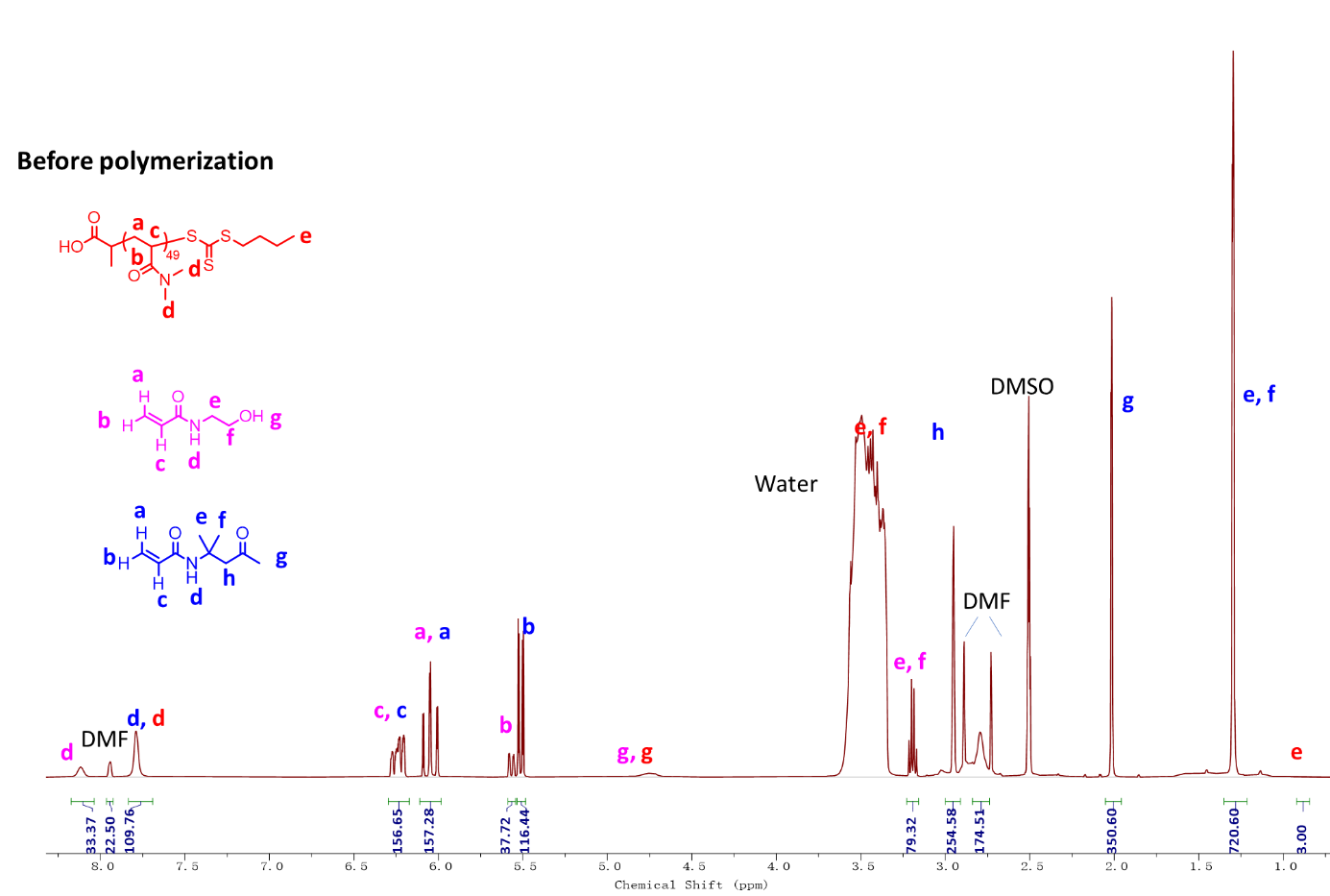

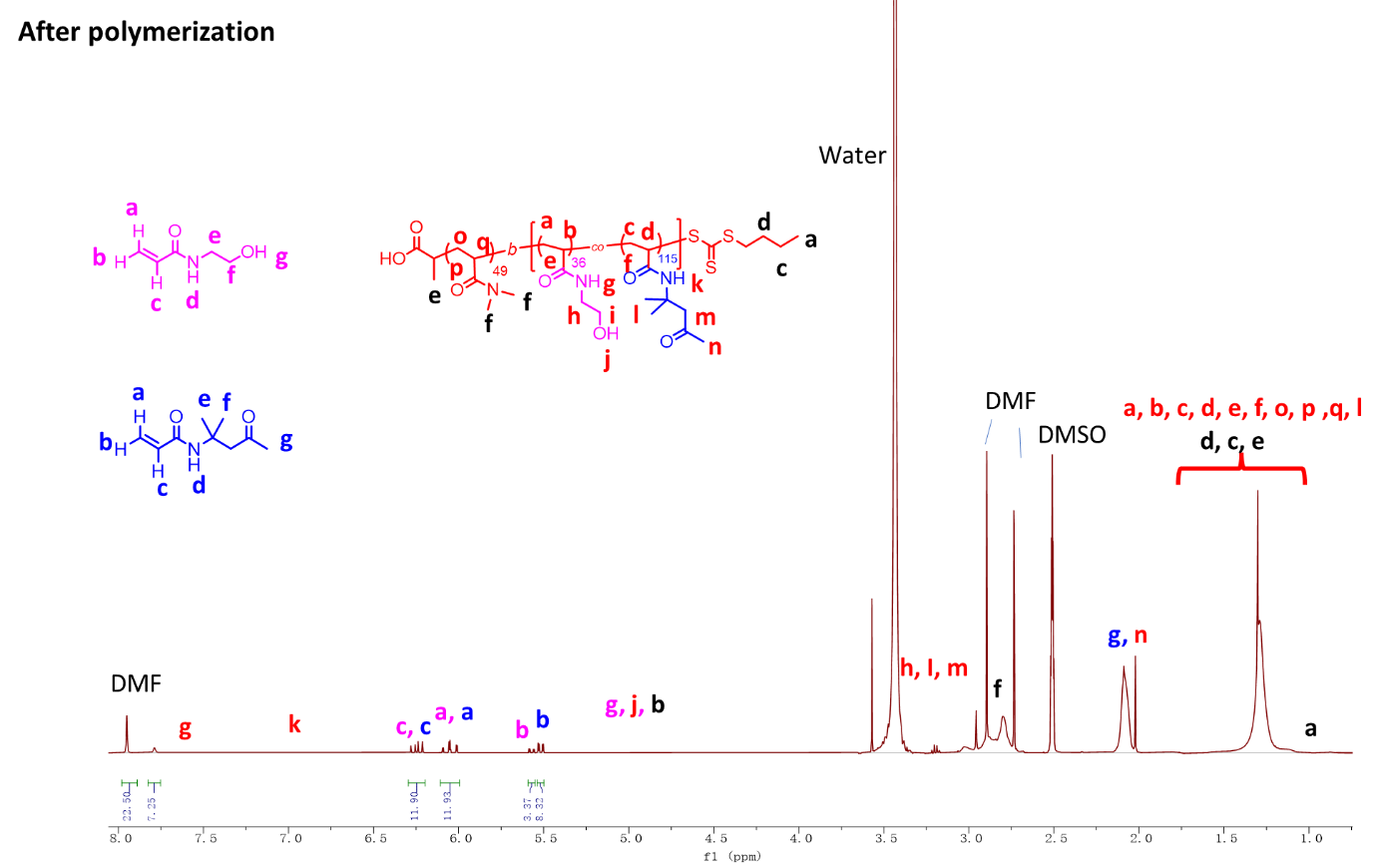


**Figure S21.** ^1^H NMR spectrum of PDMA_49_-*b*-(PHEAm_38_-*co*-PDAAm_114_) (42.8 wt%) in deuterated DMSO (400 MHz) before and after polymerization.

**Figure S22.** SEC results for PDMA_49_-*b*-(PHEAm_38_-*co*-PDAAm_114_) after chain extension.


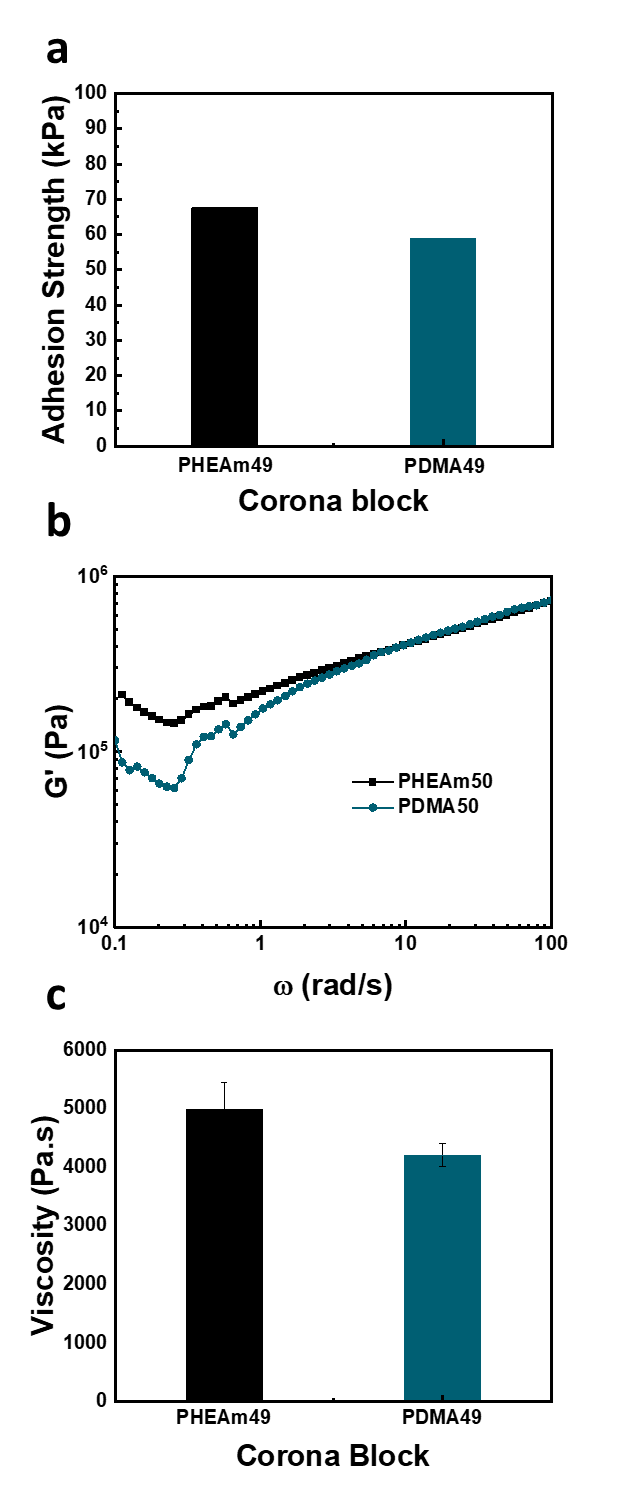


**Figure S23.** Adhesive strength, modulus, and viscosity between different corona (PHEAm49 and PDMA 49).


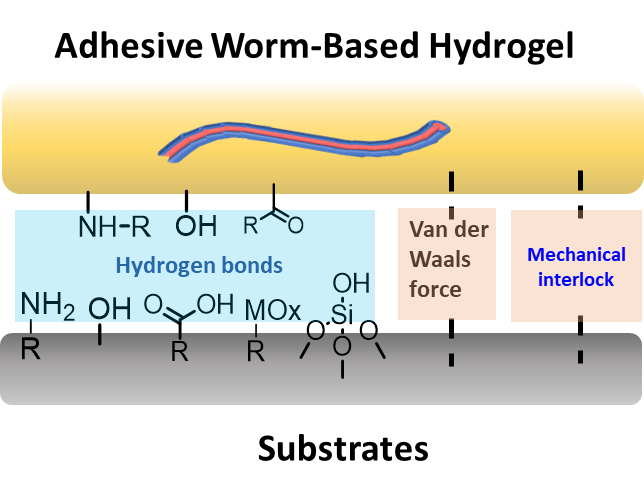


**Figure S24.** The adhesive mechanism of the worm-based hydrogel, mainly base on different hydrogen bonds, Van der Waals force and surface mechanical interlock.


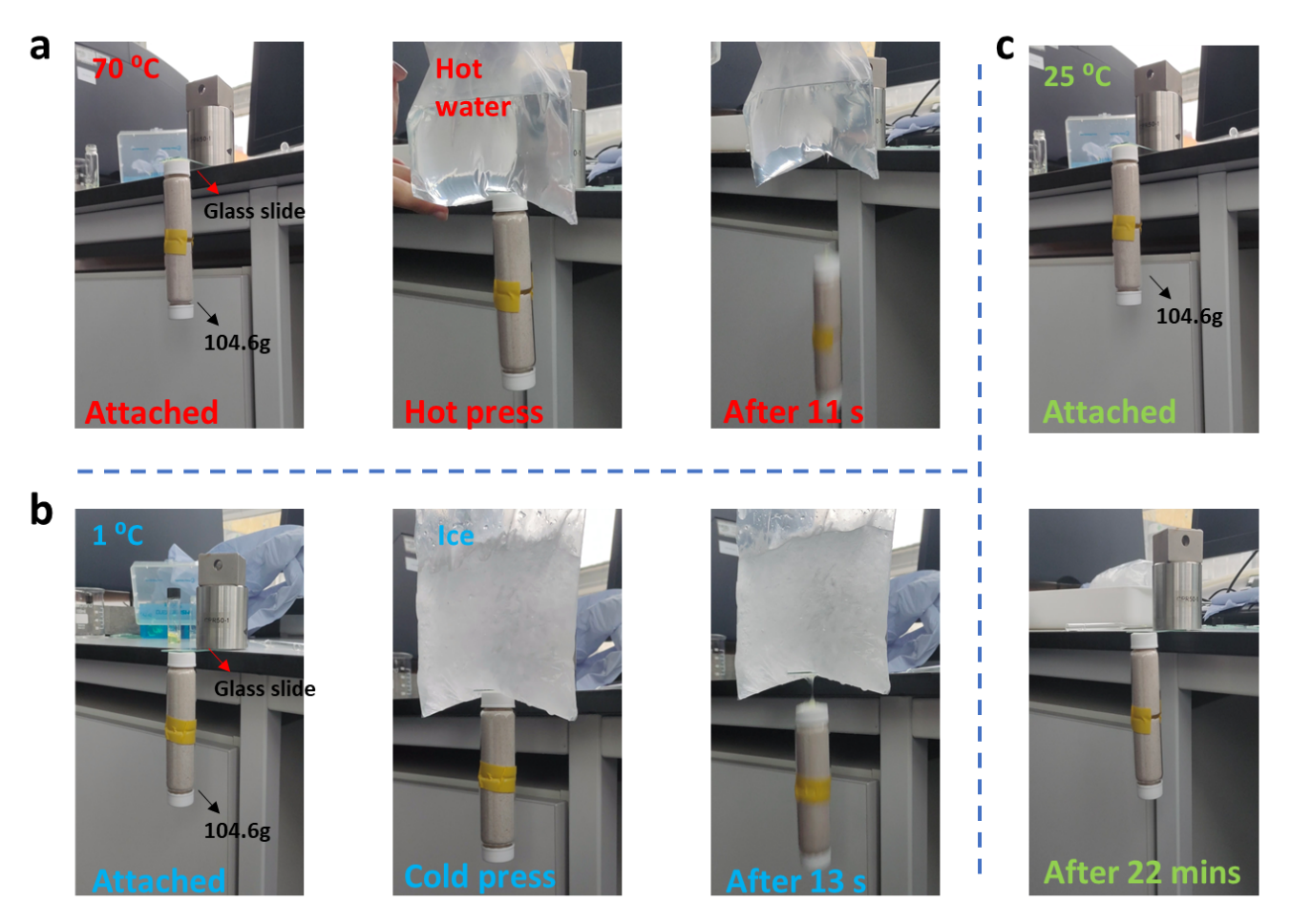


**Figure S25.** The exhibition of PHEAm_49_-*b*-(PHEAm_36_-*co*-PDAAm_115_) adhesive hydrogel adhesion under different temperatures: a) Hot press at 70 °C and detach after 11 s; b) Cold press at 1 °C and detach after 13 s; c) The adhesion remained after 22 mins at room temperature.

**Figure S26.** Cyclic durability after 100 cycles: adhesion strength after 100 attachment–detachment cycles at 1 °C.


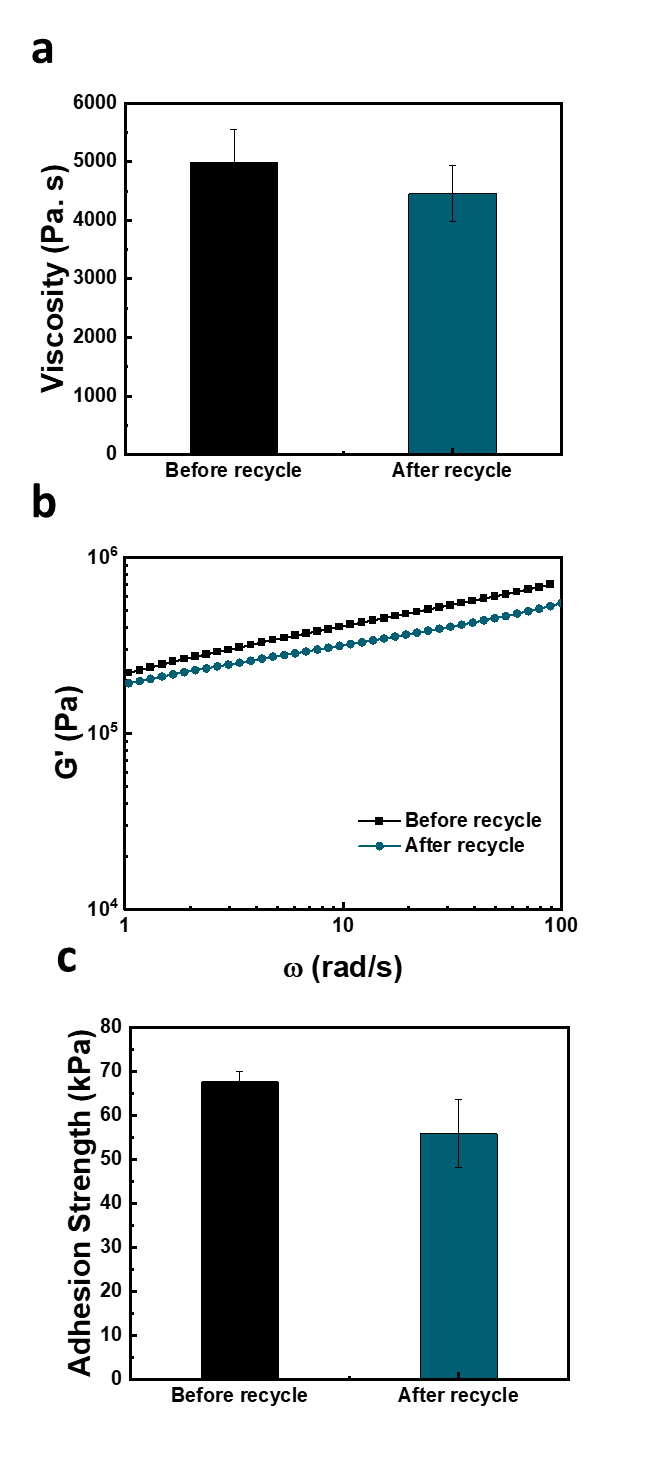


**Figure S27.** Viscosity, modulus and adhesion strength of hydrogel before recycling and after recycling.


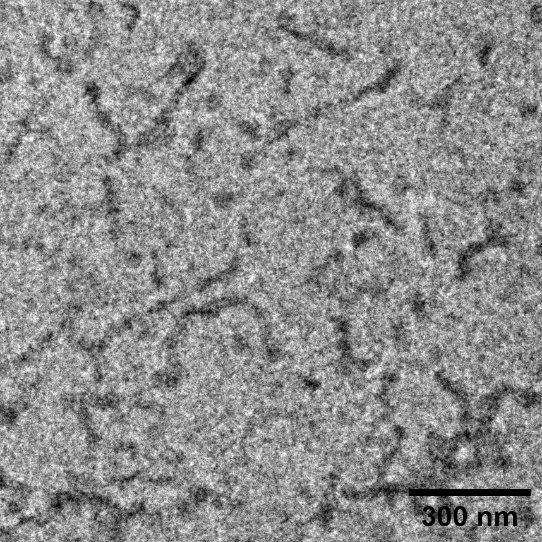


**Figure S28.** Recycled worm’s hydrogel (PHEAm_49_-*b*-(PHEAm_36_-*co*-PDAAm_115_)) TEM image at 25 °C with a similar average thickness around 25 nm.


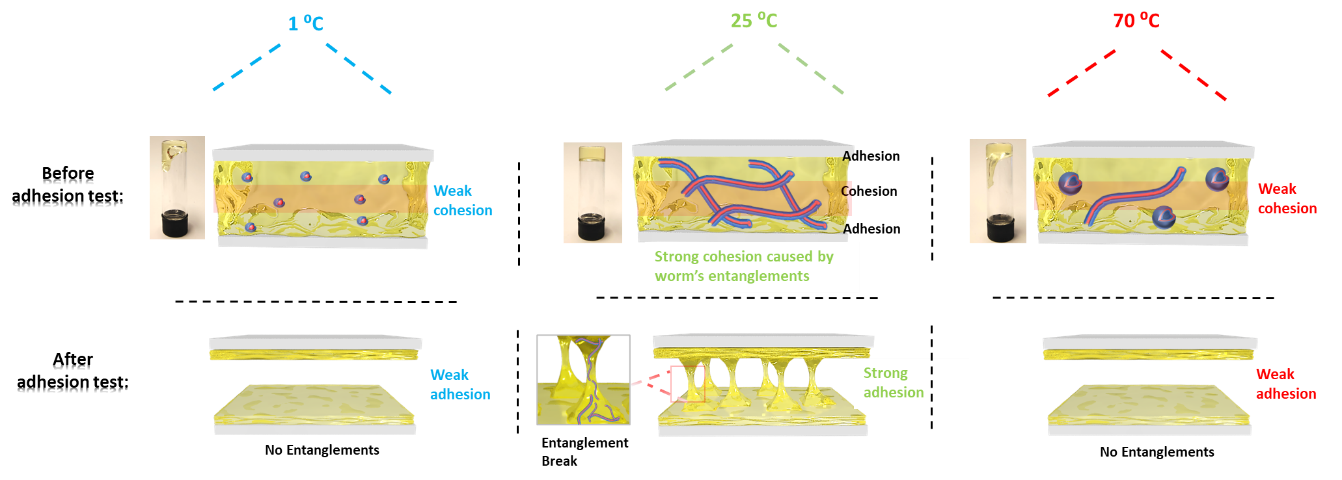


**Figure S29.** The illustration of adhesive and cohesive mechanism during the adhesive test at different temperature.

References

[1] M. Sponchioni, C. O'Brien, C. Borchers, E. Wang, M. Rivolta, N. Penfold, I. Canton, S. Armes, *Chemical Science* **2020**, *11*, 232-240; bN. J. Penfold, J. R. Whatley, S. P. Armes, *Macromolecules* **2019**, *52*, 1653-1662; cA. Krieg, C. Weber, R. Hoogenboom, C. R. Becer, U. S. Schubert, *ACS Macro Letters* **2012**, *1*, 776-779.

[2] J. Y. Rho, G. M. Scheutz, S. Häkkinen, J. B. Garrison, Q. Song, J. Yang, R. Richardson, S. Perrier, B. S. Sumerlin, *Polymer Chemistry* **2021**, *12*, 3947-3952.
